# The effect of intermittent preventive treatment for malaria with dihydroartemisinin–piperaquine on vaccine-specific responses among schoolchildren in rural Uganda (POPVAC B): a double-blind, randomised controlled trial

**DOI:** 10.1016/S2214-109X(24)00281-X

**Published:** 2024-10-16

**Authors:** Ludoviko Zirimenya, Agnes Natukunda, Jacent Nassuuna, Gyaviira Nkurunungi, Christopher Zziwa, Caroline Ninsiima, Christine Kukundakwe, Christine M Nankabirwa, Charity Katushabe, Loyce K Namusobya, Gloria Oduru, Grace Kabami, Joel Kabali, John Kayiwa, Joyce Kabagenyi, Govert J van Dam, Paul L A M Corstjens, Stephen Cose, Anne Wajja, Sarah G Staedke, Pontiano Kaleebu, Alison M Elliott, Emily L Webb, POPVAC trial team, Mirriam Akello, Florence A Akello, Hellen Akurut, Susan Amongi, Rebecca Amongin, Barbara Apule, Stephen Cose, Emmanuella Driciru, Alison M Elliott, Joyce Kabagenyi, Joel Kabali, Grace Kabami, Prossy N Kabuubi, Ayoub Kakande, Pontiano Kaleebu, Charity Katushabe, John Kayiwa, Samuel Kiwanuka, Fred Kiwudhu, Robert Kizindo, Moses Kizza, Christine Kukundakwe, Alex Mutebe, Esther Nakazibwe, Loyce Namusobya, Milly Namutebi, Christine Nankabirwa, Beatrice Nassanga, Jacent Nassuuna, Agnes Natukunda, Doreen Nayebare, Caroline Ninsiima, Ronald Nkangi, Gyaviira Nkurunungi, Denis Nsubuga, Ruth Nyanzi, Gloria Oduru, Caroline Onen, Joel Serubanja, Moses Sewankambo, Josephine Tumusiime, Pius Tumwesige, Anne Wajja, Bridgious Walusimbi, Emily L Webb, Ludoviko Zirimenya, Christopher Zziwa

**Affiliations:** a Immunomodulation and Vaccines Focus Area, Vaccine Research Theme, Medical Research Council/Uganda Virus Research Institute and London School of Hygiene & Tropical Medicine Uganda Research Unit, Entebbe, Uganda; b Department of Arbovirology, Uganda Virus Research Institute, Entebbe, Uganda; c Department of Parasitology, Leiden University Medical Center, Leiden, Netherlands; d Department of Cell and Chemical Biology, Leiden University Medical Center, Leiden, Netherlands; e Department of Infection Biology, London School of Hygiene & Tropical Medicine, London, UK; f International Statistics and Epidemiology Group, Department of Infectious Disease Epidemiology, London School of Hygiene & Tropical Medicine, London, UK; g Department of Clinical Research, London School of Hygiene & Tropical Medicine, London, UK; h Department of Global Health and Amsterdam Institute for Global Health and Development, Amsterdam University Medical Centers, Amsterdam, Netherlands

## Abstract

**Background:**

Several important vaccines differ in immunogenicity and efficacy between populations. We hypothesised that malaria suppresses responses to unrelated vaccines and that this effect can be reversed—at least partially—by monthly malaria intermittent preventive treatment (IPT) in high-transmission settings.

**Methods:**

We conducted an individually randomised, double-blind, placebo-controlled trial of the effect of malaria IPT with dihydroartemisinin–piperaquine on vaccine responses among schoolchildren aged 9–17 years in Jinja district, Uganda. Participants were recruited from two schools and did not have exposure to vaccines of interest after the age of 5 years, with the exception of human papillomavirus (HPV). Computer-generated 1:1 randomisation was implemented in REDCap. 3-day courses of dihydroartemisinin–piperaquine (dosage by weight) or placebo were administered monthly, including twice before the first vaccination. Trial participants were vaccinated with the live parenteral BCG vaccine (Serum Institute of India, Pune, India) at week 0; yellow fever vaccine (YF-17D; Sanofi Pasteur, Lyon, France); live oral typhoid vaccine (Ty21a; PaxVax, London, UK), and quadrivalent virus-like particle HPV vaccine (Merck, Rahway, NJ, USA) at week 4; and toxoid vaccines (tetanus–diphtheria; Serum Institute of India) and an HPV booster at week 28. An additional HPV vaccination at week 8 was provided to female participants older than 14 years who had not previously been vaccinated, and a tetanus–diphtheria booster was given after completion of the trial at week 52. Primary outcomes were vaccine responses at week 8 and, for tetanus–diphtheria, at week 52, and analysis was done in the intention-to-treat population. Malaria parasite prevalence at enrolment and during follow-up was determined retrospectively by PCR. The safety population comprised all randomly allocated participants. The trial was registered at the ISRCTN Registry (ISRCTN62041885) and is complete.

**Findings:**

Between May 25 and July 14, 2021, we assessed 388 potential participants for eligibility. We enrolled and randomly allocated 341 participants to the two groups (170 [50%] to dihydroartemisinin–piperaquine and 171 [50%] to placebo); 192 (56%) were female and 149 (44%) participants were male. 145 (85%) participants in the dihydroartemisinin–piperaquine group and 140 participants (82%) in the placebo group were followed up until the week 52 endpoint. At enrolment, 109 (64%) of all participants in the dihydroartemisinin–piperaquine group and 99 (58%) of 170 participants in the placebo group had malaria; this reduced to 6% or lower at all follow-up visits in the dihydroartemisinin–piperaquine group. There was no effect of dihydroartemisinin–piperaquine versus placebo on primary outcomes: BCG-specific IFNγ ELISpot response had a geometric mean ratio (GMR) of 1·09 (95% CI 0·93–1·29), p=0·28; yellow fever neutralising antibody was 1·19 (0·91–1·54), p=0·20 for plaque reduction neutralising reference tests (PRNT_50_) titres (the reciprocal of the last plasma dilution that reduced by 50%) and 1·24 (0·97–1·58), p=0·09 for PRNT_90_ titres (reciprocal of the last plasma dilution that reduced by 90%); and IgG to *Salmonella enterica* serovar Typhi O-lipopolysaccharide was 1·09 (0·81–1·46), p=0·58, HPV-16 was 0·72 (0·44–1·77), p=0·19, HPV-18 was 0·71 (0·47–1·09), p=0·11; tetanus toxoid was 1·22 (0·91–1·62), p=0·18, and diphtheria toxoid was 0·97 (0·83–1·13), p=0·72. There was some evidence that dihydroartemisinin–piperaquine reduced waning of the yellow fever response.

**Interpretation:**

IPT for malaria with dihydroartemisinin–piperaquine did not improve peak vaccine responses, despite reducing malaria prevalence. Possible longer-term effects on response waning should be further explored.

**Funding:**

UK Medical Research Council.

**Translation:**

For the Luganda translation of the abstract see Supplementary Materials section.

**Research in context**

**Evidence before this study**

On Dec 5, 2023, we searched MEDLINE, Embase, Global Health, Scopus, and Web of Science, using the Ovid interface for trials in English that assessed the effect of malaria treatment on vaccine responses, or observational studies that described the association between malaria and vaccine responses, in both humans and animals, from database inception up to Dec 5, 2023. The search strategy incorporated three search terms: “WHO-licenced vaccines” AND (“malaria infection” OR “malaria treatment”) AND “immune responses” (for full keywords see appendix 2 pp 21–22). Of the 9016 results, 35 met the inclusion criteria, of which four were animal studies and 31 were studies in humans. A wide variety of vaccines were investigated, although most studies assessed the effect of malaria on response to a single vaccine. One previous trial in humans determined that, in infants, intermittent preventive treatment of malaria with sulfadoxine**–**pyrimethamine does not affect serological responses to Essential Programme on Immunization vaccines. Observational studies assessing the association of malaria with vaccine responses found mixed results, with the strongest evidence for a negative association with malaria found for polysaccharide and tetanus toxoid vaccines.

**Added value of this study**

To our knowledge, this is the first trial assessing the impact of intermittent preventive treatment of malaria with dihydroartemisinin–piperaquine on responses to a broad set of vaccines administered to school-aged children living in a malaria-endemic area. We have shown that despite dihydroartemisinin–piperaquine being highly effective in removing and preventing malaria parasitaemia, there was no resulting impact on vaccine responses to BCG, oral typhoid, human papillomavirus, yellow fever, diphtheria, or tetanus vaccinations. There was a suggestion that dihydroartemisinin–piperaquine treatment reduced waning of the yellow fever vaccine response.

**Implications of all the available evidence**

The results demonstrate that regular intermittent preventive treatment of malaria in school-aged children does not improve or impair the immediate response to a broad range of vaccines among adolescents in a rural malaria-endemic setting. Taken together with previous observed associations between malaria and vaccine responses, we cannot rule out a role of repeated malaria exposure in immunomodulation of vaccine responses, but our results indicate that any such effects are not removed by intermittent preventive treatment treatment of malaria. Possible longer-term effects on vaccine response waning merit further study.

## Introduction

Nearly half of the global population are at risk of malaria. In 2021, there were an estimated 247 million cases and 619 000 deaths, with over 95% of both occurring in the WHO African region.^1^ Emerging insecticide and drug resistance, alongside recent health service delivery disruption due to the COVID-19 pandemic, threatens progress made towards malaria control.^1^ Intermittent preventive treatment (IPT) in school-aged children (IPTsc) was recommended by WHO in 2022 as a malaria reduction strategy in areas with moderate-to-high perennial or seasonal transmission,^1^ and IPTsc with dihydroartemisinin–piperaquine has been shown to be highly effective in Uganda.^2^

Effective vaccines are an essential tool in the control and elimination of infectious diseases. However, vaccine responses have been shown to vary between populations and to be impaired in low-income rural settings,^3^, ^4^ often the same settings where malaria is most prevalent. One hypothesis for this observation is that immunomodulation by parasitic infections, including malaria, alters vaccine responses,^5^ but evidence in humans is inconsistent. A 2010 review found evidence of an adverse effect of malaria on responses to polysaccharide vaccines, but little evidence that malaria impairs responses to protein vaccines given in multiple doses.^5^ More recently, presence of malaria at the time of vaccine response measurement was associated with decreased responses to measles immunisation in infants,^6^ but increased responses to human papillomavirus (HPV) vaccination.^7^ There is evidence that new vaccines may also be affected; in a Sierra Leone trial of the recombinant vesicular stomatitis virus–Ebola virus envelope glycoprotein vaccine (rVSVΔG-ZEBOV-GP), adults with asymptomatic malaria parasitaemia at time of vaccination had a robust immune response to the vaccine, albeit lower than seen among those without parasitaemia.^8^ Mechanisms through which malaria might affect vaccine responses are not fully understood, but effects could be due to acute immunological changes associated with fever^9^ or longer-term changes, for example in T follicular helper cell and B cell function.^10^, ^11^

If treating malaria improves vaccine responses, programmes combining parasite control with immunisation would offer an attractive and practical public health intervention for schools and communities. The Population Differences in Vaccine Responses (POPVAC) programme included three randomised controlled trials, and was designed to explore environmental exposures that might explain impaired vaccine response in rural communities in low-income settings in order to identify strategies through which vaccine effectiveness can be optimised.^12^ In the current trial (POPVAC B)^13^ we aimed to investigate the effect of malaria IPT with dihydroartemisinin–piperaquine on responses to a range of vaccines in school-aged children in a malaria-endemic area of Uganda.

## Methods

### Study design and participants

The trial protocol has been published previously.^13^ We conducted a randomised, double-blind, placebo-controlled, parallel group trial in children aged 9–17 years who were attending two rural primary schools in Jinja district, Uganda, between May 25, 2021, and Sept 6, 2022. Jinja district has malaria transmission throughout the year,^14^ with two peaks following the rainy seasons (March to May and August to October).

Participants were included if they attended one of the two selected schools (which had the highest number of pupils in the target community, enabling us to reach our sample size) in primary years 1 to 6, were aged 9–17 years, were willing to provide information on where they reside and, for female participants, if they agreed to avoid pregnancy during the trial. In our trial, sex was self-reported, with categories restricted to male and female**.** Our choice of schoolchildren and this age group was guided by the potential public health opportunity for school-delivered targeted interventions such as combined vaccination and IPT campaigns, and by previous research conducted in the same geographical area, which highlighted a notable prevalence of malaria among this specific demographic.^2^

Participants who had been vaccinated for yellow fever, oral typhoid, BCG, or tetanus and diphtheria at age 5 years or older were excluded. As HPV vaccine coverage was high in the study area, we included female participants who had been previously vaccinated, but only assessed HPV-related outcomes among participants who had not received HPV vaccination before the trial. Full inclusion and exclusion criteria are listed in appendix 2 (p 5).

**Figure 1:**
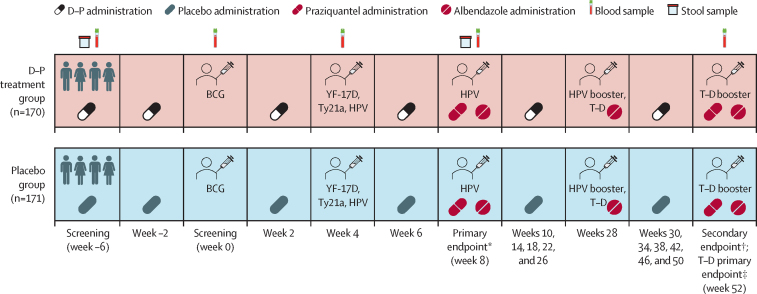
Trial schedule Samples were collected before vaccinations or anthelminthic treatment at relevant timepoints. D–P=dihydroartemisinin–piperaquine. HPV=virus-like particle human papillomavirus vaccine. T–D=tetanus–diphtheria vaccine. YF-17D=yellow fever vaccine. Ty21a=live oral typhoid vaccine. *Primary endpoint following BCG, YF-17D, Ty21a, and HPV vaccination; additionally, an HPV dose was given to previously unvaccinated girls aged 14 years or older. †Secondary endpoint following BCG, YF-17D, Ty21a, and HPV vaccination. ‡A T–D boost was given to comply with the Uganda National Expanded Program on Immunization guidelines.

Participants were randomly assigned in a 1:1 ratio to the experimental intervention group of monthly dihydroartemisinin–piperaquine (Bliss GVS Pharma, Mumbai, India) or the placebo group (figure 1). Monthly IPT with dihydroartemisinin–piperaquine has been shown to be protective in schoolchildren living in a malaria-endemic setting.^2^

All participants and their parents or guardians gave written informed assent and consent, respectively. Ethics approval was granted by the Uganda Virus Research Institute (reference GC/127/18/09/681), the London School of Hygiene and Tropical Medicine (reference 16033), the Uganda National Council for Science and Technology (reference HS2487) and the Uganda National Drug Authority (reference CTC 0117/2020). Independent trial steering and data safety monitoring committees oversaw the trial. The trial was registered at the ISRCTN Registry (ISRCTN62041885).

### Randomisation and masking

An independent statistician used randomly permuted blocks (sizes 4, 8, 6, and 10) to generate a randomisation code used to assign participants in a 1:1 ratio to receive either the dihydroartemisinin–piperaquine treament or placebo. This code was embedded into an electronic data capture system (REDCap, Vanderbilt University, Nashville, TN, USA) which was used to allocate the codes sequentially to eligible participants at enrolment. Upon random assignment, REDCap only displayed the randomisation code to be used by the study staff to identify the corresponding drug pack (containing dihydroartemisinin–piperaquine or placebo tablets). The randomisation code was kept securely by the trial statistician and made available only to those responsible for providing or preparing the trial interventions.

Dihydroartemisinin–piperaquine or placebo treatments were pre-packed by MUL staff not otherwise involved in the trial. Packs contained the maximum number of tablets of dihydroartemisinin–piperaquine or placebo that were needed (based on maximum expected weight) and were labelled with the randomisation number and trial week, and the pills were identical in appearance. Participants, investigators, and study staff involved in data validation or interpretation were masked to intervention allocation.

### Procedures

Sociodemographic and clinical details were collected from all participants at screening. Blood samples to assess baseline infections and urine samples from female participants for pregnancy testing were also collected at baseline. All screening, enrolment, and follow-up activities were done at the two schools. After enrolment, participants were seen for vaccination (trial-related vaccinations at weeks 0, 4, and 28); monthly administration of dihydroartemisinin–piperaquine or placebo; administration of praziquantel (weeks 8 and 52) and albendazole (weeks 8, 28, and 52); and obtaining blood samples (weeks 0, 4, 8, and 52) and stool samples (week 8) before and after vaccinations. Treatments were given after sampling where schedules coincided. This approach, using PCR's high sensitivity, aimed to detect low-level parasitaemia.

Participants in the dihydroartemisinin–piperaquine treatment group received two 3-day courses of oral dihydroartemisinin–piperaquine 1 month apart, before the first immunisation, followed by monthly 3-day treatments thereafter. Each dose was calculated based on the participant's weight as per the manufacturer's guidelines. Dihydroartemisinin–piperaquine tablets (40 mg dihydroartemisinin and 320 mg piperaquine phosphate) were administered once a day for 3 consecutive days. The standard group received placebo tablets, calculated by weight as per the study drug, as routine preventive malaria treatment in schools is not yet required by Uganda Ministry of Health policy. All treatments were directly observed by an investigator.

Malaria standard care included bednet provision to minimise malaria exposure for all participants. For all participants who presented with a fever (temperature >37**·**5°C recorded via thermometer in the armpit) at the timepoint of dihydroartemisinin–piperaquine or placebo administration, a malaria Rapid Diagnostic Test (Global Diagnostics, Tamil Nadu, India) was done. For participants who tested positive for malaria, dihydroartemisinin–piperaquine or placebo administration was omitted, and treatment with artemether–lumefantrine was offered by the trial staff per Ugandan national guidelines.

Albendazole and praziquantel were provided to all participants after primary and secondary endpoint samples were collected, as per Ugandan national guidelines to manage nematode and trematode infections, respectively.

The vaccination schedule (figure 1) consisted of three main vaccination days at weeks 0, 4, and 28. Trial participants were vaccinated with the live parenteral BCG vaccine (Serum Institute of India, Pune, India; 0·1 mL intradermally, right upper arm) at week 0; yellow fever vaccine (YF-17D; Sanofi Pasteur, Lyon, France; 0·5 mL intramuscularly, left upper arm); live oral typhoid vaccine (Ty21a; PaxVax, London, UK; one capsule per day taken every other day for 3 days), and quadrivalent virus-like particle HPV vaccine (Merck, Rahway, NJ, USA; 0·5 mL intramuscularly, left upper arm) at week 4; and toxoid vaccines (tetanus–diphtheria; Serum Institute of India; 0·5 mL intramuscularly, left upper arm) and an HPV booster at week 28. An additional HPV vaccination at week 8 was provided to female participants older than 14 years who had not previously been vaccinated, and a tetanus–diphtheria booster was given after completion of the trial at week 52, in accordance with the national Expanded Programme on Immunisation (EPI) routines. Strategies to mitigate risk of participant dropout and ensure robust follow-up are described in appendix 2 (p 13).

The primary outcomes were BCG-specific IFNγ responses 8 weeks post-BCG vaccination; YF-17D-neutralising antibody titres at 4 weeks post-YF-17D vaccination; *Salmonella enterica* serovar Typhi (henceforth *S* Typhi) O-lipopolysaccharide (O:LPS)-specific IgG concentration at 4 weeks post-Ty21a vaccination; HPV type-16 and type-18 L1 protein-specific IgG concentration at 4 weeks post-HPV priming vaccination; and tetanus and diphtheria toxoid-specific IgG concentration at 24 weeks post-tetanus–diphtheria vaccination. Primary outcome assays were conducted at week 8 (for tetanus–diphtheria at week 52). Pre-vaccination responses were assessed at week 28 for tetanus–diphtheria; pre-vaccination responses for all other vaccines were assessed at week 0.

Our original protocol^13^ specified assessment of baseline levels of tetanus and diphtheria toxoid-specific IgG concentration at week 8, before tetanus–diphtheria vaccination, and the primary outcome for tetanus–diphtheria at week 32, 4 weeks after the immunisation. However, this schedule was amended based on protocol changes during the COVID-19 pandemic, and also for financial considerations. Thus, the primary outcome for tetanus–diphtheria was assessed 24 weeks post-vaccination, at week 52 of the trial. The target sample size was also modified in this COVID-19-related protocol amendment (see Statistical Analysis section).

We assessed BCG-specific IFNγ responses using freshly isolated peripheral blood mononuclear cells (PBMCs) and a Human IFNγ (ALP) ELISpot Flex kit (Mabtech, Stockholm, Sweden). Assay details are documented in appendix 2 (pp 14–15). We report results as spot-forming units (SFUs) per million PBMCs, calculated sequentially by first, subtracting mean SFUs of unstimulated assay wells from mean SFUs of duplicate BCG-stimulated wells; and second, correcting for the number of PBMCs (300 000) per well. Samples that had more than 83·3 SFUs per million PBMCs in the unstimulated well were considered invalid and not included in the final analysis.

Plasma neutralising antibodies against yellow fever virus were assessed using plaque reduction neutralising reference tests (PRNTs, appendix 2 pp 15–16). We report PRNT_50_ and PRNT_90_ titres, defined as the reciprocal of the last plasma dilution that reduced by 50% or 90%, respectively, the number of virus plaques infected by 100 plaque forming units per 0·1 mL of the reference 17D virus preparation. Plasma HPV-16-specific and HPV-18-specific IgG antibodies were measured using an ELISA, adapted from a method employed by Miller and colleagues.^15^ *S* Typhi O:LPS-specific IgG levels were quantified by ELISA, using standards from the Oxford Vaccine Centre Biobank (Oxford, UK). Anti-diphtheria and anti-tetanus IgG levels were also determined by ELISA, using WHO reference preparations and standards. Detailed methods are given in appendix 2 (p 18).

Planned secondary outcomes were assessment of response waning by measurement of the primary outcomes described above but at week 52 (for all vaccinations except tetanus and diphtheria) and area under the curve (AUC) combining week 8 and week 52 responses; the proportion of participants with protective neutralising antibodies for yellow fever, protective IgG levels for tetanus toxoid, and seroconversion rates for Ty21a at 4 weeks post the corresponding immunisation (24 weeks for tetanus toxoid); and effects of the intervention on priming versus boosting for HPV only, comparing outcomes at 4 weeks after the first dose with outcomes at week 52. Participants with PRNT_50_ titres of ten or greater following YF-17D vaccination were considered seropositive.^16^ Tetanus toxoid-specific IgG levels greater than or equal to 0·1 international units (IUs) per mL post-tetanus–diphtheria vaccination^17^ were considered protective. Seroconversion following Ty21a vaccination was defined as a 4-fold or greater increase in *S* Typhi O:LPS-specific IgG over baseline.^18^ *Plasmodium falciparum* infection status was also assessed retrospectively by PCR on stored blood samples collected on immunisation days and at week 52. The safety population comprised all randomly allocated participants.

### Statistical analysis

The original target sample size of 640 participants was based on the assumption that the primary analysis would be done among participants with malaria infection at enrolment (assumed to be ≥60% of those enrolled).^13^ However, recruitment was halted at a sample size of 341 due to the COVID-19-related nationwide lockdown and school shutdowns in Uganda. This circumstance was discussed with the Trial Steering Committee who highlighted that since all children were likely to be at similar risk of malaria during the study, it would be more appropriate for the primary analysis to include all participants regardless of infection status at baseline. This mitigated the effect of the unavoidable reduction in sample size. The new sample size of 341 participants was determined to give over 80% power to detect absolute mean differences of 0·11 log_10_ to 0·21 log_10_ in primary outcomes, assuming SDs of 0·3 log_10_ to 0·6 log_10_, at 5% significance level and allowing for 20% loss to follow-up. The original and updated power estimates are shown in appendix 2 (p 3).

Baseline characteristics of participants, and the percentage of participants receiving each dihydroartemisinin–piperaquine dose, placebo dose, and each vaccination were summarised by trial group. The prevalence of malaria PCR positivity at each timepoint was compared between trial groups using χ^2^ tests.

Analysis was done by intention to treat (ITT), so that participants were included in the group to which they were randomly assigned, regardless of the number of dihydroartemisinin–piperaquine or placebo doses received. The ITT analysis group for each vaccine was further defined as all participants who received each vaccine, and for whom vaccine immune response was measured, regardless of the number of doses of dihydroartemisinin–piperaquine or placebo taken. Participants were included in the ITT analysis group regardless of adherence to the study protocol or completion of follow-up visits. Data for participants with missing vaccine immune responses were excluded.

For BCG and YF-17D responses, we excluded participants who did not receive the corresponding vaccine from the analysis. For Ty21a response, we excluded participants who received none of the three Ty21a vaccine doses. For HPV responses, we excluded from the analysis data from females who had received any doses of HPV vaccination before enrolment in POPVAC B or who received an additional dose at week 8.

Primary outcomes were log_10_ transformed and compared between trial groups using unpaired Student's *t* tests with results back-transformed to give geometric mean ratios (GMRs) and 95% CIs. For the secondary outcomes of response waning of YF-17D, Ty21a, and HPV, we compared responses at week 52, and AUC from week 8 and week 52 responses, using the same approach as described for the primary outcomes. Protective immunity outcomes for yellow fever, Ty21a, and tetanus were summarised as proportions and compared between trial groups as differences in proportions and corresponding 95% CIs. The effect of dihydroartemisinin–piperaquine versus placebo on priming versus boosting for HPV was assessed among those who had received both HPV doses (weeks 4 and 28) in the trial, using a mixed effects linear regression model for HPV response at weeks 8 and 52 and including an interaction term between timepoint and trial group. No adjustments for covariates were made in the primary analyses, but in exploratory analyses we investigated the effect of controlling for the corresponding vaccine response at baseline. In a planned subgroup analysis we assessed whether the effect of dihydroartemisinin–piperaquine versus placebo on primary outcomes differed by sex, using linear regression of log-transformed outcomes and including an interaction term between trial group and participant sex. Analyses and data visualisation were done in Stata version 17 and GraphPad version 9.0.0.

### Role of the funding source

The funder and sponsor of the trial had no role in trial design, data collection, data analysis, data interpretation, or writing of the report.

## Results

**Figure 2:**
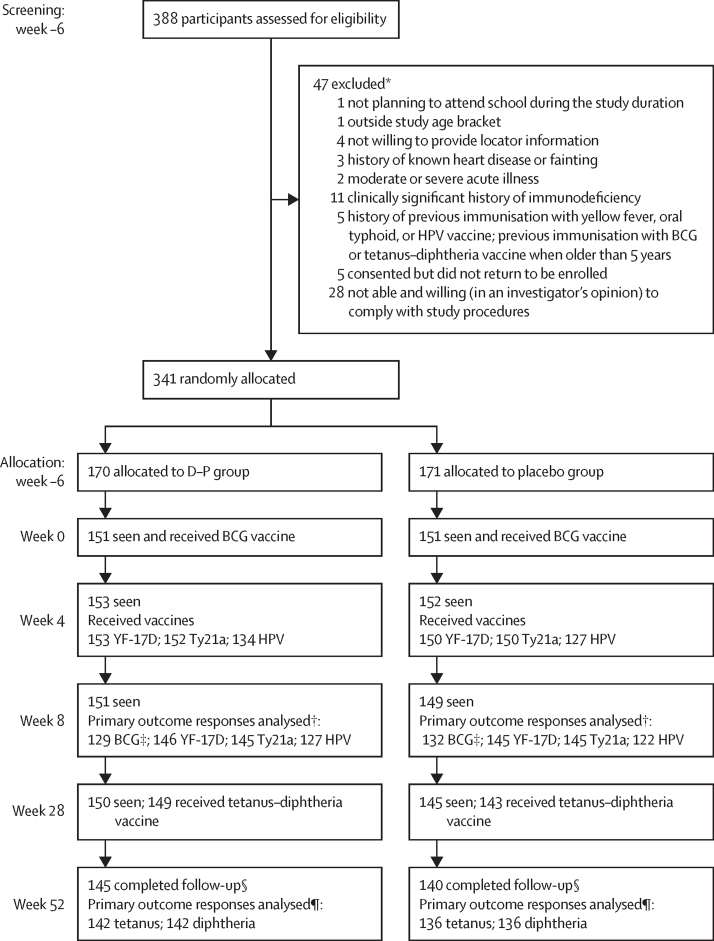
Trial profile D–P=dihydroartemisinin**–**piperaquine. YF-17D=yellow fever vaccine. Ty21a=live oral typhoid vaccine. HPV=virus-like particle human papillomavirus vaccine. PBMCs=peripheral blood mononuclear cells. SFU=spot-forming units. *Some participants had multiple reasons for exclusion †Reasons for not being included in the final analysis included no sample at the primary endpoint, or the sample was available, but the participant did not receive the correctly allocated vaccine. ‡BCG INFγ gamma ELISpot assay—samples that had more than 83·3 SFUs per a million PBMCs in the unstimulated well were considered invalid and not included in the final analysis. §In the D–P group, 25 participants did not complete follow-up, while 31 did not complete follow-up in the placebo group, totalling 56 participants. The reasons for withdrawal were being lost to follow-up (n=31), became pregnant during follow-up (n=1) and withdrawal of consent (n=24). ¶Reasons for not being included in the final analysis were no sample at week 52, or the sample was available but participant did not receive a tetanus–diphtheria vaccine at week 28.

Between May 25 and July 14, 2021, we assessed 388 potential participants for eligibility. We enrolled and randomly allocated 341 participants to the two groups (170 [50%] to dihydroartemisinin–piperaquine and 171 [50%] to placebo). 145 (85%) participants in the dihydroartemisinin–piperaquine group and 140 participants (82%) in the placebo group were followed up until the week 52 endpoint. The last participant completed follow-up on Sept 6, 2022. The trial profile is shown in figure 2.

149 (44%) participants were male and 192 (56%) were female. Median age was 13 years (IQR 11–13). At enrolment, 109 (64%) of all participants in the dihydroartemisinin–piperaquine group and 99 (58%) of 170 participants in the placebo group had malaria, testing positive for *P falciparum* by PCR. 56 patients did not complete follow-up (25 [15%] in the dihydroartemisinin–piperaquine group and 31 [18%] in the placebo group; figure 2). The reasons for withdrawal were loss to follow-up (n=31), becoming pregnant during follow-up (n=1) and withdrawal of consent (n=24). Baseline characteristics of participants are shown in table 1.

**Table 1:** Baseline demographic and clinical characteristics

		**Dihydroartemisinin–piperaquine group (n=170)**	**Placebo group (n=171)**
Age in years, median (IQR)		13 (11–13)	13 (11–14)
Sex			
	Male	77 (45%)	72 (42%)
	Female	93 (55%)	99 (58%)
BMI, median (IQR)		17·5 (16·1–18·9)	17·4 (16·1–18·7)
BCG scar present		108 (64%)	113 (66%)
School			
	Muguluka Church of Uganda primary school	121 (71%)	124 (73%)
	Namalere Church of Uganda primary school	49 (29%)	47 (27%)
Malaria infection at baseline, PCR positive for *Plasmodium falciparum*		109/170 (64%)	99/170* (58%)
Helminth infections			
	*Schistosoma mansoni*, CAA ≥30 pg/mL	2/170 (1%)	6/171 (4%)
	*S mansoni*, PCR positive	41/168 (24%)	39/170 (23%)
	*Necator americanus*, PCR positive	24/168 (14%)	17/170 (10%)
	*Strongyloides stercoralis*, PCR positive	5/168 (3%)	2/170 (1%)

Data are n (%) unless otherwise stated. CAA=circulating anodic antigen.

* One patient did not have PCR data available.

**Figure 3:**
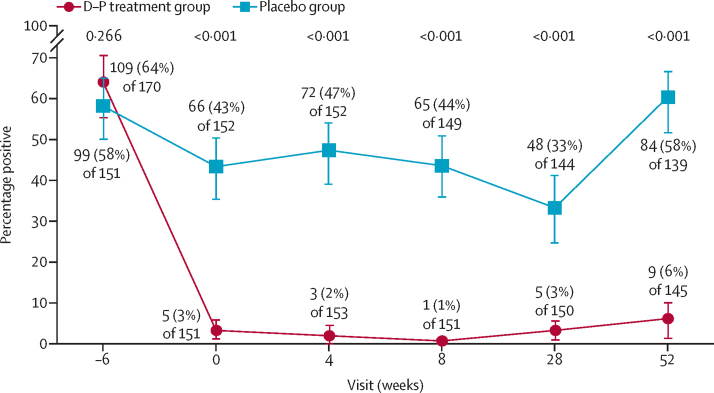
Malaria infection status by visit Malaria prevalence at each timepoint was compared between trial groups using χ^2^ tests. Linear mixed models were fitted to compare malaria prevalence (p<0·0001) between trial groups, from week –6 to week 52. The numbers of participants with PCR results available at each timepoint were 340 [99%] of 342 at week –6; 303 [100%] of 303 at week 0; 305 [100%] of 305 at week 4, 300 [100%] of 300 at week 8, 294 [100%] of 295 at week 28, and 284 [100%] of 284 at week 52. D–P=dihydroartemisinin–piperaquine.

Dihydroartemisinin–piperaquine and placebo coverage rates per individual daily dose ranged from 73% to 99% in the dihydroartemisinin–piperaquine group and 79% to 100% in the placebo group across the 15 treatment rounds (appendix 2 pp 3–4); the mean dose coverage was 91% in the dihydroartemisinin–piperaquine group and 92% in the placebo group. Malaria prevalence reduced to 6% or lower at all follow-up visits in the dihydroartemisinin–piperaquine group (figure 3), while the mean malaria prevalence at follow-up visits in the placebo group was 41% when weighted for percentage of participants with PCR.

151 (89%) of 170 and 151 (88%) of 171 participants assigned to the dihydroartemisinin–piperaquine and placebo group, respectively, received the first vaccination with BCG at week 0. At week 4, vaccine uptake in the dihydroartemisinin–piperaquine versus the placebo group for each vaccine respectively was 153 (90%) of 170 versus 150 (88%) of 171 for YF-17D; 152 (89%) of 170 versus 150 (88%) of 171 for the first dose of Ty21a; and 134 (91·2%) of 147 versus 127 (87%) of 146 for HPV. At week 28, 149 (88%) of 170 enrolled participants in the dihydroartemisinin–piperaquine group and 143 (84%) of 171 in the placebo group received the tetanus–diphtheria vaccination. Full details on vaccine uptake are shown in appendix 2 (p 4). Pre-vaccination vaccine-specific responses were balanced between the trial groups (appendix 2 p 5).

There was no significant effect of dihydroartemisinin–piperaquine compared with placebo treatment on any vaccine response at the primary outcome timepoints: BCG-specific IFNγ ELISpot response had a GMR of 1·09 (95% CI 0·93–1·29), p=0·28; yellow fever neutralising antibody was 1·19 (0·91–1·54), p=0·20 for PRNT_50_ titres and 1·24 (0·97–1·58), p=0·09 for PRNT_90_ titres; and IgG to *S* Typhi O-lipopolysaccharide (O-LPS) was 1·09 (0·81–1·46), p=0·58, HPV-16 was 0·72 (0·44–1·77), p=0·19, HPV-18 was 0·71 (0·47–1·09), p=0·11, tetanus toxoid was 1·22 (0·91–1·62), p=0·18, and diphtheria toxoid was 0·97 (0·83–1·13), p=0·72 (table 2 and appendix 2 p 10). Sensitivity analysis excluding 19 participants in the placebo group who received artemether–lumefantrine during the study resulted in similar findings to the primary analysis (appendix 2 pp 9, 11).

**Table 2:** Effect of dihydroartemisinin–piperaquine versus placebo on vaccine responses

		**n**	**Geometric mean (SE)**	**Geometric mean ratio (95% CI)**	**p value**
**Primary endpoint analysis**					
BCG-specific IFNγ (8 weeks post-vaccination), SFUs per 1 million PBMCs					
	Dihydroartemisinin–piperaquine	129	294·23 (1·06)	1·09 (0·93–1·29)	0·28
	Placebo	132	269·21 (1·06)	ref	..
Yellow fever PRNT_50_ titres (4 weeks post-vaccination)					
	Dihydroartemisinin–piperaquine	146	2021·53 (1·10)	1·19 (0·91–1·54)	0·20
	Placebo	145	1706·41 (1·10)	ref	..
Yellow fever PRNT_90_ titres (4 weeks post-vaccination)					
	Dihydroartemisinin–piperaquine	146	183·78 (1·10)	1·24 (0·97–1·58)	0·09
	Placebo	145	148·48 (1·09)	ref	..
*S* Typhi O:LPS-specific IgG (4 weeks post-vaccination), EU/mL					
	Dihydroartemisinin–piperaquine	145	300·90 (1·11)	1·09 (0·81–1·46)	0·58
	Placebo	145	276·95 (1·11)	ref	..
HPV-16-specific IgG (4 weeks post-vaccination), EU/mL*					
	Dihydroartemisinin–piperaquine	83	78·66 (1·19)	0·72 (0·44–1·17)	0·19
	Placebo	83	210·47 (1·19)	ref	..
HPV-18-specific IgG (4 weeks post-vaccination), EU/mL*					
	Dihydroartemisinin–piperaquine	83	386·50 (1·17)	0·71 (0·47–1·09)	0·11
	Placebo	83	543·01 (1·16)	ref	..
Tetanus toxoid-specific IgG (24 weeks post-vaccination), IU/mL					
	Dihydroartemisinin–piperaquine	142	6·17 (1·11)	1·22 (0·91–1·62)	0·18
	Placebo	136	5·08 (1·11)	ref	..
Diphtheria toxoid-specific IgG (24 weeks post-vaccination), IU/mL					
	Dihydroartemisinin–piperaquine	142	2·41 (1·06)	0·97 (0·83–1·14)	0·72
	Placebo	136	2·48 (1·06)	ref	..
**Secondary endpoint analysis**					
BCG-specific IFNγ (52 weeks post-vaccination), SFUs per 1 million PBMCs					
	Dihydroartemisinin–piperaquine	125	100·31 (1·07)	0·89 (0·73–1·10)	0·29
	Placebo	118	112·22 (1·08)	ref	..
Yellow fever PRNT_50_ titres (week 52, 48 weeks post-vaccination)					
	Dihydroartemisinin–piperaquine	138	997·92 (1·11)	1·10 (0·83–1·45)	0·52
	Placebo	134	910·74 (1·11)	ref	..
Yellow fever PRNT_90_ titres (week 52, 48 weeks post-vaccination)					
	Dihydroartemisinin–piperaquine	138	143·54 (1·09)	1·31 (1·04–1·65)	0·02
	Placebo	134	109·46 (1·08)	ref	..
*S* Typhi O:LPS-specific IgG (week 52, 48 weeks post-vaccination), EU/mL					
	Dihydroartemisinin–piperaquine	137	100·66 (1·10)	1·10 (0·86–1·41)	0·43
	Placebo	134	91·30 (1·08)	ref	..
HPV-16-specific IgG (week 52, 48 weeks post-vaccination), EU/mL *					
	Dihydroartemisinin–piperaquine	76	353·21 (1·14)	0·82 (0·57–1·19)	0·29
	Placebo	76	430·94 (1·14)	ref	..
HPV-18-specific IgG (week 52, 48 weeks post-vaccination), EU/mL*					
	Dihydroartemisinin–piperaquine	76	749·91 (1·12)	0·76 (0·55–1·06)	0·10
	Placebo	76	987·90 (1·13)	ref	..

EU=ELISA unit. HPV=human papillomavirus. IU=international unit. O:LPS=O-lipopolysaccharide. PMBC=peripheral blood mononuclear cells. PRNT_50_=plaque reduction neutralising reference tests, for the reciprocal of the last plasma dilution that reduced by 50%. PRNT_90_=plaque reduction neutralising reference tests, for the reciprocal of the last plasma dilution that reduced by 90%. SFU=spot-forming units. *S* Typhi=*Salmonella enterica* serovar Typhi.

* Analysis population for HPV vaccine is participants who had not received HPV vaccination before the trial.

For secondary outcome analyses at week 52, there was no significant difference in vaccine responses between the trial groups for the BCG-specific IFNγ ELISpot response (GMR 0·89 [95% CI 0·73–1·10], p=0·29); yellow fever PRNT_50_ titres (1·10 [0·83–1·45], p=0·52); and IgG to *S* Typhi O-LPS (1·10 [0·86–1·41], p=0·43), HPV-16 (0·82 [0·57–1·19], p=0·29), HPV-18 (0·76 [0·55–1·06], p=0·10; table 2 and appendix 2 p 10). However, participants in the dihydroartemisinin–piperaquine group had, on average, higher yellow fever PRNT_90_ titres (1·31 [1·04–1·65], p=0·02), and a higher AUC for this outcome measured between weeks 8 and 52 (1·33 [1·09–1·62], p=0·01). A similar, but not statistically significant, effect was seen for waning of the response assessed by AUC for yellow fever PRNT_50_ titres (1·20 [0·97–1·49], p=0·09). For all other vaccines, there was no effect of dihydroartemisinin–piperaquine on AUC between week 8 and week 52 (appendix 2 p 5).

Proportions with protective immunity at 4 weeks post the corresponding vaccination (24 weeks for tetanus–diphtheria) were not significantly different between the two trial groups (appendix 2 p 6). Furthermore, there was no difference in the effect of dihydroartemisinin–piperaquine treatment on HPV vaccine-specific priming or boosting doses from week 8 to week 52 (p_interaction_ 0·58 and 0·51, respectively; appendix 2 p 6).

In planned subgroup analyses by sex, there was some suggestion that dihydroartemisinin–piperaquine versus placebo reduced HPV-18 responses in male participants (GMR 0·76 [95% CI 0·57–1·01]) but not in female participants (1·26 [0·68–2·33]), although the test for interaction was not significant (p_interaction_=0·15; appendix 2 pp 7, 11). Both HPV-16 and HPV-18 responses were substantially lower in male participants than in female participants following administration of HPV vaccination in this trial, despite exclusion of females who had already been vaccinated, and similar antibody levels in males and previously unvaccinated females at enrolment. There was no evidence of effect modification for responses to any other trial vaccines.

One serious adverse event was reported, a road traffic accident judged unrelated to the trial intervention. Adverse events and their frequencies were as expected, and similar between trial groups (appendix 2 pp 7–8).

## Discussion

In this randomised, placebo-controlled, double-blind trial, we aimed to make a comprehensive assessment of the influence of effective preventive treatment for malaria on the immune response to unrelated vaccines. We found that dihydroartemisinin–piperaquine was highly effective in achieving a sustained reduction in malaria prevalence among schoolchildren aged 9–17 years in rural Uganda, but that there were no consistent effects, either positive or negative from our two-sided statistical approach, on their immune responses to BCG, yellow fever, oral typhoid, HPV, tetanus, or diphtheria vaccinations. This was contrary to our hypothesis that preventive malaria treatment would improve vaccine-induced immune responses.

Our findings align with previous studies showing no effect of malaria on protein-based vaccines,^5^ but appear to contrast with findings that have suggested a role for malaria in the impairment of responses to several other vaccines.^19^, ^20^ There are several possible explanations. First, although malaria prevalence is high in many rural low-income settings where vaccine responses are impaired, malaria might not be the primary driver of these effects; most published evidence on the association of malaria with vaccine responses is observational, and observed effects might have resulted from confounding by other variables that could influence vaccine responses, such as cytomegalovirus infection^21^ or malnutrition.^22^ Second, most studies investigating the effect of malaria on vaccine responses have examined associations with current clinical or asymptomatic malaria, rather than the effects of malaria treatment or prevention. It is possible that there are persistent effects of previous malaria exposure on the immune system that are not reversed by IPT. Third, throughout the trial, participants in both trial groups who presented with fever and tested positive for malaria were treated with artemether–lumefantrine, which could explain the modest reduction in malaria prevalence seen in the placebo group during weeks 0–28, although this could also be a consequence of increased treatment-seeking after random allocation, or other behavioural changes as a result of being included in a trial. Our trial provides evidence on the effect of controlling asymptomatic malaria, but not of treating clinical malaria episodes, since the latter was done in both trial groups. Our approach of treating symptomatic malaria participants with artemether–lumefantrine might also have led to our overestimating the true positivity rate in the placebo group, since PCR can remain positive several weeks after successful treatment of malaria, although a sensitivity analysis excluding participants in the placebo group who received treatment for malaria during the study did not substantially alter vaccine responses. Our findings are consistent with trial data that IPT with sulfadoxine–pyrimethamine in infants does not affect serological responses to Expanded Program on Immunization vaccines.^23^ While we acknowledge that the choice of vaccines used in our trial might not capture the entire spectrum of immunogenicity and efficacy variations across different populations, we believe that the selected vaccines represent a relevant and comprehensive portfolio for assessing vaccine responses in the study context of adolescents in rural malaria-endemic settings in LMICs.

Although there were no effects of dihydroartemisinin–piperaquine on primary outcomes, dihydroartemisinin–piperaquine was associated with increased yellow fever PRNT_90_ titres at week 52, and in AUC analysis incorporating responses at weeks 8 and 52, suggesting a possible positive effect of malaria removal on the waning response to yellow fever vaccination. Earlier work comparing yellow fever neutralising antibody titres between adults who had been vaccinated previously in Entebbe, Uganda versus Lausanne, Switzerland, suggested much greater waning of response in Ugandan compared with Swiss participants.^24^ Further exploration of effects of malaria, and other environmental exposures, on differences in vaccine response waning between settings may be important for informing policy on booster schedules.

HPV vaccination is now recognised as a key strategy for prevention of cervical cancer and other genital cancers, and it has recently been proposed that a single dose in adolescence is sufficient—greatly simplifying programme logistics.^25^ We were concerned that a single priming dose might be more susceptible to environmental immunomodulators than the multi-dose regimen.^26^ However, we found no evidence of a difference in the effect of IPTsc on priming and boosting responses for HPV, suggesting that malaria parasites will not impair responses to a single-dose HPV strategy.^25^ Data from Brown and colleagues suggests that malaria exposure may actually increase responses to HPV-16 and HPV-18 vaccine^7^ and, aligned to this, we observed a somewhat lower HPV-18 response in the dihydroartemisinin–piperaquine-treated male participants, compared with the placebo group. HPV responses were also generally lower among male participants than female participants, as has previously been observed.^27^

Our trial had strengths and limitations. Malaria was highly prevalent in the study population and dihydroartemisinin–piperaquine was extremely effective in reducing malaria prevalence throughout the trial period. Hence our trial was well positioned to determine if there was an effect of removal of malaria infection at either time of vaccination, or time of response measurement, on vaccine-specific immune response. Trial enrolment was balanced between male and female participants, enabling us to perform sex-based subgroup analyses for primary outcomes. Since previous work has suggested that malaria could have different effects on different types of vaccines, we included a comprehensive range of vaccines that would be beneficial to adolescents including live and inert, parenteral and oral, and priming and boosting vaccinations. However, our schedule did not include some types of vaccine, such as viral vectored or mRNA vaccines. We used a randomised placebo-controlled trial design, with participants and trial personnel masked to the intervention, thus minimising risk of bias in assessing outcomes.

Due to COVID-19-related lockdowns and prolonged school closures we recruited a smaller sample size than originally planned, hence statistical power was somewhat reduced. However, this was mainly offset by the decision, taken in conjunction with the Trial Steering Committee, to include all randomly assigned participants in the primary analysis population, and also by lower than anticipated loss to follow-up. Since HPV vaccination had recently been rolled out in the study area, this necessitated the inclusion of some girls who had previously been vaccinated and who we excluded from assessment of HPV vaccine responses, reducing power for HPV response outcomes. In addition, the amendment to measure the tetanus–diphtheria primary outcome 24 weeks post vaccination instead of 4 weeks was not optimal and could have restricted our ability to demonstrate impact of IPT on tetanus–diphtheria vaccine responses. The long-term effects of malaria treatment on vaccine responses could not be evaluated as participants were only followed up for 52 weeks. While our study focused on assessing the immediate impact of malaria IPT on vaccine responses among adolescents in a rural malaria-endemic setting, we acknowledge that the immunomodulatory effects of malaria might be influenced by various exposures, including host, other parasite, and environmental factors. Furthermore, giving dihydroartemisinin–piperaquine at the time of primary immunisations might have a varying effect on the vaccine responses, although current evidence suggests otherwise.^23^ The trial was able to provide robust evidence on relatively short-term effects of removing malaria infections, since the first dose of dihydroartemisinin–piperaquine was given 6 weeks before receiving the first vaccination. However, we cannot determine the impact of longer-term previous malaria exposure using this approach. Further work will use antibody measurements to investigate this.^28^ In summary, our results demonstrate that regular preventive treatment of asymptomatic malaria does not improve or impair the immediate response to a comprehensive range of vaccines among schoolchildren aged 9–17 years in a rural malaria-endemic setting. Further research could explore programming of the immune system by life-course malaria exposure which might be difficult to reverse with short-term preventive treatment.

### POPVAC trial team

### Contributors

### Equitable partnership declaration

### Data sharing

The de-identified individual participant data that underlie the results reported in this article are stored in a non-publicly available repository (London School of Hygiene & Tropical Medicine [LSHTM] Data Compass), together with a data dictionary. Data are available on request. Researchers who would like to access the data may submit a request through LSHTM Data Compass, detailing the data requested, the intended use for the data, and evidence of relevant experience and other information to support the request. The request will be reviewed by the Principal Investigator in consultation with the Medical Research Council/Uganda Virus Research Institute (MRC/UVRI) and LSHTM data management committee, with oversight from the UVRI and LSHTM ethics committees. In line with the MRC policy on data sharing, there will have to be a good reason for turning down a request. Patient Information Sheets and consent forms specifically referenced making anonymised data available and this has been approved by the relevant ethics committees. Researchers given access to the data will sign data sharing agreements which will restrict the use to answering pre-specified research questions. We plan to disseminate the results through an impactful peer-reviewed journal, stakeholder workshops, existing partnerships and collaborations, and traditional and digital media channels to ensure they inform policy and practice effectively.

## Declaration of interests

GN reports funding from the EDCTP2 programme supported by the EU, and from the Wellcome Trust. AME, PK and SC report funding from the UK Medical Research Council (MRC) for conduct of the study; AME reports funding from UK National Institute of Health and care Research (NIHR), Science for Africa Foundation, and DELTAS Africa, outside the submitted work. AME and SC further report support from the Serum Institute of India and Emergent BioSolutions; AME reports support from Uganda National Expanded Program on Immunization; SC reports support from Bliss GVS Pharma, India, for conduct of the study. All other authors declare no competing interests.

## Supplementary Materials

Luganda translation of the abstract

Supplementary appendix 2

Equitable Partnership Declaration
