# Supplementary material for: The effect of intermittent preventive treatment for malaria with dihydroartemisinin–piperaquine on vaccine-specific responses among schoolchildren in rural Uganda (POPVAC B): a double-blind, randomised controlled trial
Source: Lancet Glob Health. 2024 Oct 16;12(11):e1838–48. doi: 10.1016/S2214-109X(24)00281-X (PMC11483247; doi:10.1016/S2214-109X(24)00281-X)
Supplement: Luganda translation of the abstract [file mmc1.pdf]

THE LANCET
Global Health

Supplementary appendix 1

This translation in Luganda was submitted by the authors and we reproduce it as supplied. It has not been peer reviewed. The Lancet's editorial processes have only been applied to the original in English, which should serve as reference for this manuscript.

Ekiwandiiko kino ekikyuse mu lulimi Oluganda kyaweereddwayo abawandiisi b'ennyini era tukifulumizza nga bwe baakituwadde. Tekinnakubaganyizibwako birowoozo abakugu abalala. Okusunsula kwa Lancet kukoleddwa ku ekyo eky'Olungereza kyokka nga bwe kyafulumizibwa era kye kirina okujulizibwako ku kiwandiiko kino.

Supplement to: Zirimenya L, Natukunda A, Nassuuna J, et al. The effect of intermittent preventive treatment for malaria with dihydroartemisinin–piperaquine on vaccine-specific responses among schoolchildren in rural Uganda (POPVAC B): a double-blind, randomised controlled trial. *Lancet Glob Health* 2024; **12:** e1838–48.

Mu bufunze

Ennyanjula

Engeri omubiri gye gwanukulamu ku ddagala erigema endwadde ekyukakyuka okusinziira ku bantu ab'enjawulo. Twakola okuteebereza nti omusujja gw'ensiri gulemesa omubiri okuwulira eddagala erigema eritalina kakwate ku linnaalyo era okulemesa kuno kusobola okukyusibwa, waakiri ekitundu nga tuyita mu kukozesa eddagala eriziyiza okukwatibwa obulwadde bw'omusujja gw'ensiri erya buli mwezi mu bifo ebirina omusujja guno nga mungi.

Enkola

Twakola okugezesa okw'okweroboza omuntu nga tewali nsonga yonna egobererwa, nga omwetabi n'omunoonyereza tekuli amanyi bujjanjabi bw'aweereddwa, n'okugezesa okulimu ebibinja okuli ekiweebwa eddagala ettuufu n'ekirala ekiweebwa ekyefanaanyiriza eddagala ly'omusujja gw'ensiri naye nga temuli ddagala okulaba ekiva mu kujjanjaba musujja gw'ensiri n'eddagala lya dihydroartemisinin-piperaquine (DP) ku ngeri omubiri gye gweyisamu ku ddagala erigema mu baana abasoma (emyaka 9-17) mu disitulikiti y'e Jinja, Uganda. Abeetabi baggyibwa mu masomero abiri era baali tebakubwangako ku ddagala ligema erikozesebwa oluvannyuma lw'emyaka etaano egy'obukulu ng'oggyeeko HPV. Enteekateeka efulumizibwa kompyuta ey'okweroboza mu kusunsula ku 1:1 ye yateekebwa mu nkola mu REDCap, Ddoozi za DP (doozi okusinziira ku buzito) nga za nnaku ssatu, oba ekyefaanaanyiriza eddagala ery'ekiguumaaza lyabaweebwa buli mwezi, nga mw'otwalidde okulibawa emirundi ebiri ng'okugemebwa okusooka tekunnabaawo. Abeetabi baafuna eddagala erigema elya BCG (Serum Institute of India, Pune, India) ku wiiki zero, omusujja gw'enkaka (YF-17D; Sanofi Pasteur, Lyon, France), omusujja gwo mu byenda (Ty21a; PaxVax, London, UK), HPV esooka (Merck, Rahway, NJ, USA) ku wiiki 4, ne HPV booster, tetanus-diphtheria ku wiiki 28. Okugema okulala okwa HPV mu wiiki 8 kwaweebwa abawala abeetabyeemu nga abasukka mu emyaka 14 abaali tebagemeddwa emabegako. Eddagala erigema tetanus–diphtheria lyaweebwa abetabi oluvannyuma lw'okumaliriza okunonyereza ku wiiki 52. Ebikulu ebyali bisuubirwa gwe mubiri okubaako ne bwe gweyisa eri eddagala erigema ku wiiki 8, ne ku tetanus-diphtheria ku wiiki 52, era okwekenneenya kwakolebwa mu bantu abaali bagenderera okujjanjabwa. Okubaawo kw'omusujja gw'ensiri mu kuyingizibwa

kwabeetabi ne mu kwekeneenyezebwa okwaddako kwakeberebwa luvannyuma nga tukozesa PCR. Omusomo guno gwawandiisibwa mu ISRCTN Registry (ISRCTN62041885) era gwaggwa.

Ebyavaamu

Wakati wa 25 Ogwokutaano ne 14 Ogw’omusanvu 2021, tweekebejja abasobola okwetabamu 388 mu kakungunta. Twayingiza abeetabi 341 mu ngeri etali ya kyekubiira nebateekebwa mu bibinja bibiri, (170 [50%] ku DP ate 171 [50%] ku kyefananyiriza eddagala ly’ekiguumaaza); 192 (56%) baali bawala ate nga 149 (44%) baali balenzi. Abeetabi 145 (85%) mu kibinja ekifuna DP n’abeetabi 140 (82%) mu kibinja ku kyefananyiriza eddagala ly’ekiguumaaza baalondoolwa okutuuka ku wiiki 52. Mu kuyingizibwa, 109 (64%) ku beetabi mu kibinja kya DP ne 99 (58%) mu kibinja kye ekyefananyiriza eddagala ely’ekiguumaaza baalina omusujja gw’ensiri; bano baakendeera okutuuka ku 6% oba wansi ko ku nkyala ezaddako ku luuyi lwa DP. Tewaaliwo njawulo ereetebwawo DP na njawulo ku kyefanaanyiriza ddagala ly’ekiguumazza mu byavaamu: okuddamu kw’abaserikale b’omubiri eri BCG kwalina omugeragerany0 gwa geometric mean ratio (GMR) ogwa 1·09 (95% CI 0·93–1·29), p=0·28; abaserikale b’omubiri okuddamu omusujja gw'enkaka kwalina GMR ya 1·19 (0·91–1·54), p=0·20 ku PRNT50 ne 1·24 (0·97–1·58), p=0·09 ku PRNT90; era abaserikale b’omubiri okwanukula ku kugema typhoid kwalina GMR ya 1·09 (0·81–1·46), p=0·58, HPV-16 yali 0·72 (0·44–1·77), p=0 ·19, HPV-18 yali 0·71 (0·47-1·09), p=0·11; abaserikale b’omubiri okwanukula ku kugema tetanus kwalina GMR ya 1·22 (0·91–1·62), p=0·18, ate diphtheria yali 0·97 (0·83–1·13), p=0·72. Waaliwo obukakafu obumu nti DP yakendeeza ku kukendeera kw’okwanukula kw’omusujja gw'enkaka.

Entaputa

Obujjanjabi obw’okuziyiza omusujja gw’ensiri n’eddagala lya DP tebwayongera ku maanyi g’enneeyisa y’omubiri eri eddagala erigema newankubadde bwasobola okukendeeza obulungi omusujja gw’ensiri. Ebiyinza okuva mu ngeri y’omubiri gye gweyisamu ku ddagala erigema oluvanyuma lwe bbanga eggwanvu egenda yeeyongera okuvumbeera era kirina okwongera okunoonyerezebwako.

Abataddemu ensimbi

UK Medical Research Council (MR/R02118X/1).
