# Supplementary material for: The effect of intermittent preventive treatment for malaria with dihydroartemisinin–piperaquine on vaccine-specific responses among schoolchildren in rural Uganda (POPVAC B): a double-blind, randomised controlled trial
Source: Lancet Glob Health. 2024 Oct 16;12(11):e1838–48. doi: 10.1016/S2214-109X(24)00281-X (PMC11483247; doi:10.1016/S2214-109X(24)00281-X)
Supplement: Supplementary appendix 2 [file mmc2.pdf]

THE LANCET
Global Health

Supplementary appendix 2

This appendix formed part of the original submission and has been peer reviewed. We post it as supplied by the authors.

Supplement to: Zirimenya L, Natukunda A, Nassuuna J, et al. The effect of intermittent preventive treatment for malaria with dihydroartemisinin–piperaquine on vaccine-specific responses among schoolchildren in rural Uganda (POPVAC B): a double-blind, randomised controlled trial. *Lancet Glob Health* 2024; **12:** e1838–48.

SUPPLEMENTARY INFORMATION FOR

The effect of intermittent preventive treatment for malaria with dihydroartemisinin-piperaquine on vaccine-specific responses among schoolchildren in rural Uganda: results of the POPVAC B randomised, controlled trial

Ludoviko Zirimenya,* Agnes Natukunda,* Jacent Nassuuna,* Gyaviira Nkurunungi,* Christopher Zziwa, Caroline Ninsiima, Christine Kukundakwe, Christine M Nankabirwa, Charity Katushabe, Loyce K Namusobya, Gloria Oduru, Grace Kabami, Joel Kabali, John Kayiwa, Joyce Kabagenyi, Govert J van Dam, Paul LAM Corstjens, Stephen Cose, Anne Wajja, Sarah G Staedke,¶ Pontiano Kaleebu, Alison M Elliott,# Emily L Webb# **and the POPVAC trial team†**

Immunomodulation and Vaccines Focus Area, Vaccine Research Theme, Medical Research Council/Uganda Virus Research Institute and London School of Hygiene and Tropical Medicine (MRC/UVRI and LSHTM) Uganda Research Unit, Entebbe, Uganda *(L Zirimenya MPH, A Natukunda MSc, J Nassuuna MSc, G Nkurunungi PhD, C Zziwa BSc, C Ninsiima BSc, C Kukundakwe BSc, C Nankabirwa RN, C Katushabe Dip Clin Med, LK Namusobya BSWSA, G Oduru BSc, G Kabami, J Kabali BSc, J Kabagenyi MSc, S Cose PhD, A Wajja MSc, Prof. P Kaleebu PhD, Prof. AM Elliott MD)*

Department of Arbovirology, Uganda Virus Research Institute, Entebbe, Uganda (*J Kayiwa MSc*)

Department of Parasitology, Leiden University Medical Center, Leiden, The Netherlands (*GJ van Dam PhD*)

Department of Cell and Chemical Biology, Leiden University Medical Center, Leiden, The Netherlands (*PLAM Corstjens PhD*)

Department of Infection Biology, London School of Hygiene and Tropical Medicine, London, United Kingdom (*G Nkurunungi PhD*)

International Statistics and Epidemiology Group, Department of Infectious Disease Epidemiology, London School of Hygiene and Tropical Medicine, London, United Kingdom (*A Natukunda MSc, Prof. EL Webb PhD*)

Department of Clinical Research, London School of Hygiene and Tropical Medicine, London, United Kingdom (*L Zirimenya MPH, A Wajja MSc, S Cose PhD, Prof. SG Staedke PhD, Prof. AM Elliott MD*)

Department of Global Health and Amsterdam Institute for Global Health and Development, Amsterdam University Medical Centers, Amsterdam, Netherlands (*A Wajja MSc*)

*Joint first authors

#Joint senior authors

¶Current address: Department of Vector Biology, Liverpool School of Tropical Medicine, Liverpool, UK

†Trial team members listed at the end of this article

Correspondence to: Dr Gyaviira Nkurunungi, Immunomodulation and Vaccines Focus Area, Vaccine Research Theme, MRC/UVRI and LSHTM Uganda Research Unit, P.O. Box 49, Entebbe, Uganda.
Gyaviira.Nkurunungi@mrcuganda.org

Supplementary tables

Table S1. Original and updated power estimates, at 5% significance level and allowing for 20% loss to follow-up

Standard deviation (log_{10})	Log_{10} difference						
	**0.08**	**0.10**	**0.12**	**0.14**	**0.16**	**0.18**	**0.20**
Original power estimates: 192 DP vs 192 placebo (malaria infected only, assumed prevalence 60%)							
0.3	65%	83%	94%	98%	>99%	>99%	>99%
0.4	42%	59%	75%	87%	94%	98%	99%
0.5	29%	42%	56%	69%	80%	88%	94%
0.6	21%	31%	42%	53%	65%	75%	83%
Updated power estimates; 170 DP vs 171 placebo (all participants, regardless of malaria infection)							
0.3	59%	78%	91%	97%	99%	>99%	>99%
0.4	38%	54%	69%	82%	91%	96%	98%
0.5	26%	38%	51%	64%	75%	84%	91%
0.6	20%	28%	39%	48%	59%	69%	78%
Cells highlighted in grey correspond to >80% power.							

Table S2. DP/placebo administration at each visit pre and post BCG vaccination at week 0.

Time point	Dosage	Placebo, n/N (%)	DP, n/N (%)
6 weeks pre	Dose 1	164/171 (95.9)	155/170 (91.2)
	Dose 2	150/171 (87.7)	142/170 (83.5)
	Dose 3	135/171 (79.0)	124/170 (72.9)
2 weeks pre	Dose 1	159/171 (93.0)	156/170 (91.8)
	Dose 2	154/171 (90.1)	146/170 (85.9)
	Dose 3	152/171 (88.9)	146/170 (85.9)
2 weeks post	Dose 1	149/171 (87.1)	154/170 (90.6)
	Dose 2	149/171 (87.1)	150/170 (88.2)
	Dose 3	146/171 (85.4)	147/169 (87.0)
6 weeks post	Dose 1	143/156 (91.7)	154/164 (93.9)
	Dose 2	139/156 (89.1)	149/164 (90.9)
	Dose 3	138/156 (88.5)	149/164 (90.9)
10 weeks post	Dose 1	148/171 (86.6)	147/170 (86.5)
	Dose 2	145/171 (84.8)	141/170 (82.9)
	Dose 3	145/171 (84.8)	146/170 (85.9)
14 weeks post	Dose 1	148/169 (87.6)	150/170 (88.2)
	Dose 2	145/169 (85.8)	146/170 (85.9)
	Dose 3	147/169 (87.0)	145/170 (85.3)
18 weeks post	Dose 1	143/168 (85.1)	142/164 (86.6)
	Dose 2	141/168 (83.9)	137/164 (83.5)
	Dose 3	137/168 (81.6)	137/163 (84.1)
22 weeks post	Dose 1	136/140 (97.1)	135/141 (95.7)
	Dose 2	135/140 (96.4)	132/141 (93.6)
	Dose 3	134/140 (95.7)	128/141 (90.8)
26 weeks post	Dose 1	142/146 (97.3)	149/150 (99.3)
	Dose 2	137/146 (93.8)	145/150 (96.7)
	Dose 3	134/146 (91.8)	144/150 (96.0)

30 weeks post	Dose 1	144/144 (100.0)	147/152 (96.7)
	Dose 2	143/144 (99.3)	143/152 (94.1)
	Dose 3	143/144 (99.3)	147/152 (96.7)
34 weeks post	Dose 1	131/136 (96.3)	131/134 (97.8)
	Dose 2	130/136 (95.6)	130/134 (97.0)
	Dose 3	134/138 (97.1)	132/137 (96.4)
38 weeks post	Dose 1	139/141 (98.6)	136/142 (95.8)
	Dose 2	134/141 (95.0)	133/142 (93.7)
	Dose 3	134/141 (95.0)	126/142 (88.7)
42 weeks post	Dose 1	138/145 (95.2)	145/147 (98.6)
	Dose 2	135/145 (93.1)	143/147 (97.3)
	Dose 3	133/145 (91.7)	139/147 (94.6)
46 weeks post	Dose 1	138/142 (97.2)	142/146 (97.3)
	Dose 2	137/142 (96.5)	136/146 (93.2)
	Dose 3	137/142 (96.5)	130/145 (89.7)
50 weeks post	Dose 1	135/141 (95.7)	137/143 (95.8)
	Dose 2	131/140 (93.6)	136/141 (96.5)
	Dose 3	127/137 (92.7)	136/141 (96.5)

Table S3. Number of participants vaccinated in each trial arm.

	**Week 0 BCG**	**Week 4 HPV, YF-17D and Ty21a**	**Week 8 HPV***	**Week 28 HPV**	**Week 28 Td**	**Week 52 Td****
	n/N(%)§	n/N(%)§	n/N(%)§	n/N(%)§	n/N(%)§	n/N(%)§
DP arm	151/170 (88.8)		2/2 (100.0)	94/170 (55.3)	149/170 (87.6)	149/170 (87.6)
Placebo arm	151/171 (88.3)		1/1 (100.0)	94/171 (55.0)	143/171 (83.6)	143/171 (83.6)

	**Week 4 HPV¥**	**Week 4 YF-17D**	**Week 4*** Ty21a**		
	**n/N(%)§**	**n/N(%)§**	**n/N(%)§**		
			**Dose 1**	**Dose 2**	**Dose 3**
DP arm	134/147 (91.2)	153/170 (90.0)	152/170 (89.4)	147/170 (86.5)	147/170 (86.5)
Placebo arm	127/146 (87.0)	150/171 (87.7)	150/171 (87.7)	142/171 (83.0)	145/171 (84.8)

§ n/N (%) Number vaccinated/Number expected at the time of vaccination (Percent who were vaccinated); at each visit.

¥Week 4 HPV was only given to participants who did not have prior full HPV vaccination.

*Among girls aged >14 years who had not received HPV vaccine before this trial

**Td booster at week 52 provided as a service

***145/170(85.3%) participants in the DP arm received all three doses of oral typhoid vaccination.

***141/171(82.4%) participants in the placebo arm received all three doses of oral typhoid vaccination.

***152/170(89.4%) participants in the DP arm received at least one dose of oral typhoid vaccination.

***150/171(87.7%) participants in the placebo arm received at least one dose of oral typhoid vaccination.

Table S4. Baseline vaccine responses

Antigen specific responses	n	DP arm (N=170) Median IQR)	n	Placebo arm (N=171) Median (IQR)	P value
BCG-specific IFN-ɣ (SFUs/1 million PBMCs)*	129	78.3 (45.0- 131.7)	126	85.0 (45.0-155.0)	0.47
Yellow fever $PRNT_{50}$ titres*	151	5.0 (5.0-5.0)	152	5.0 (5.0-5.0)	0.74
Yellow fever $PRNT_{90}$ titres*	151	5.0 (5.0-5.0)	152	5.0 (5.0-5.0)	0.97
S. typhi O:LPS-specific IgG (EU/ml)*	151	75.2 (36.8-161.9)	152	74.6 (39.7-126.6)	0.56
HPV-16-specific IgG (EU/ml)*	86	4.7 (2.9-7.6)	87	4.6 (2.8-7.3)	0.98
HPV-18-specific IgG (EU/ml)*	86	66.1 (41.8-108.8)	87	75.6 (41.4-108.0)	0.62
Tetanus toxoid-specific IgG (IU/ml)[#]	150	0.09 (0.06-0.28)	145	0.08 (0.04-0.17)	0.04
Diphtheria toxoid-specific IgG (IU/ml)[#]	150	0.14 (0.05-0.37)	145	0.15 (0.07-0.29)	0.99

*Baseline responses measured at week 0. [#]Baseline responses measured at week 8.

Table S5. Differences in vaccine responses between DP and placebo arms (area under the curve for weeks 8 to 52)

Trial arm	n	GM (AUC) SE	GMR (AUC) (95% CI)	P value
**BCG-specific IFN-ɣ**				
DP	110	1035(1.06)	0.99 (0.85-1.16)	0.92
Placebo	108	1044(1.06)	Ref.	
**Yellow fever $PRNT_{50}$ titres**				
DP	133	95789(1.08)	1.20 (0.97-1.49)	0.09
Placebo	130	79604(1.08)	Ref.	
**Yellow fever $PRNT_{90}$ titres**				
DP	133	9498(1.08)	**1.33 (1.09-1.62)**	**0.01**
Placebo	130	7150(1.07)	Ref.	
***S. Typhi* O:LPS-specific IgG**				
DP	132	10679(1.11)	1.07 (0.80-1.42)	0.67
Placebo	130	10024(1.11)	Ref.	
**HPV-16-specific IgG**				
DP	72	11737(1.16)	0.78 (0.52-1.18)	0.24
Placebo	73	15029(1.16)	Ref.	
**HPV-18-specific IgG**				
DP	72	29781(1.15)	0.726 (0.50-1.06)	0.10
Placebo	73	41024(1.14)	Ref.	

AUC: area under the curve; CI: confidence interval; GM: geometric mean; GMR: geometric mean ratio; SE: standard error

Table S6. Proportions with protective immunity between trial arms at 4 weeks post the corresponding vaccination (24 weeks for TT)

	**DP**	**Placebo**	**Proportion difference for protection (95% CI)**	**P value***
	n/N (%)	n/N (%)		
Protective neutralizing antibody (Yellow fever $PRNT_{50}$ titres)	146/146(100.0)	145/145(100.0)	-	-
Protective neutralizing antibody (Yellow fever $PRNT_{90}$ titres)	143/146(98.0)	144/145(99.3)	-0.014 (-0.040-0.013)	0.317
Protective IgG levels (Tetanus toxoid-specific IgG)	139/142(97.9)	135/136(99.3)	-0.014 (-0.041-0.014)	0.335
Seroconversion rates (*S. Typhi* O:LPS-specific IgG)	43/136(31.6)	45/137(32.9)	-0.012 (-0.123-0.099)	0.828

*P values reported from chi square test
Reference for the difference in proportion is the placebo arm.

Table S7. Priming versus boosting: comparison of HPV responses between trial arms after priming and boosting doses.

DP: n=72; Placebo n=73	**GMR (95%) CI**	**P value**	**Interaction p value[1]**
**HPV-16-specific IgG-DP**			0.58
Week 52 vs week 8	4.63 (3.40-6.30)	0.00	
**HPV-16-specific IgG-Placebo**			
Week 52 vs week 8	4.10 (3.02-5.57)	0.00	
**HPV-18-specific IgG-DP**			0.51
Week 52 vs week 8	2.09 (1.63-2.68)	0.00	
**HPV-18-specific IgG-Placebo**			
Week 52 vs week 8	1.86 (1.45-2.38)	0.00	

[1]Interaction test to assess whether the boosting effect differed by trial arm. Includes those who received 2 doses of HPV during the study and had responses at weeks 8 and 52.

Table S8. Comparison of outcomes at the primary outcome time point by trial arm, stratified by sex.

	Females				Males				
**Arm**	**n**	**GM(SE)**	**GMR (95% CI)**	**P value**	**n**	**GM(SE)**	**GMR (95% CI)**	**P value**	**Interaction p**
**BCG- specific IFN-γ (8 weeks post-vaccination)**									
**DP**	71	307.09 (1.08)	1.14 (0.92-1.41)	0.216	58	279.21 (1.10)	1.03 (0.80-1.34)	0.80	0.55
**Placebo**	78	268.69 (1.08)	Ref.		54	269.96 (1.09)	Ref.		
**Yellow fever $PRNT_{50}$ titres (4 weeks post-vaccination)**									
**DP**	81	2033.81 (1.13)	1.13 (0.81-1.56)	0.467	65	2006.34(1.17)	1.27 (0.82-1.97)	0.28	0.66
**Placebo**	85	1803.15 (1.12)	Ref.		60	1578.16(1.17)	Ref.		
**Yellow fever $PRNT_{90}$ titres (4 weeks post-vaccination)**									
**DP**	81	173.00 (1.12)	1.16 (0.86-1.57)	0.328	65	198.15 (1.17)	1.34 (0.89-2.02)	0.16	0.57
**Placebo**	85	149.01 (1.11)	Ref.		60	147.75 (1.15)	Ref.		
**S. Typhi O:LPS-specific IgG (4 weeks post-vaccination)**									
**DP**	80	257.05 (1.15)	0.97 (0.65-1.44)	0.870	65	365.27 (1.17)	1.24 (0.80-1.93)	0.33	0.41
**Placebo**	85	265.71 (1.15)	Ref.		60	293.70 (1.17)	Ref.		
**HPV-16-specific IgG (4 weeks post-vaccination)**									
**DP**	62	958.57 (1.29)	1.33 (0.66-2.68)	0.418	65	47.47 (1.13)	0.80 (0.58-1.12)	0.19	0.20
**Placebo**	62	719.45 (1.28)	Ref.		60	59.10 (1.12)	Ref.		
**HPV-18-specific IgG (4 weeks post-vaccination)**									
**DP**	62	3564.89 (1.26)	1.26 (0.68-2.33)	0.467	65	253.58 (1.12)	0.76 (0.57-1.01)	0.06	0.15
**Placebo**	62	2840.29 (1.23)	Ref.		60	333.15 (1.09)	Ref.		
**Tetanus toxoid-specific IgG (24 weeks post-vaccination)**									
**DP**	77	5.30 (1.14)	1.34 (0.97-1.84)	0.074	65	7.40 (1.18)	1.02 (0.62-1.68)	0.94	0.35
**Placebo**	80	3.96 (1.11)	Ref.		56	7.25 (1.22)	Ref.		
**Diphtheria toxoid-specific IgG (24 weeks post-vaccination)**									
**DP**	77	2.28 (1.07)	0.93 (0.76-1.13)	0.453	65	2.57 (1.09)	1.03 (0.80-1.32)	0.85	0.53
**Placebo**	80	2.46 (1.07)	Ref.		56	2.51 (1.10)	Ref.		

Table S9A. Adverse events

Summary

	DP arm N=170		Placebo arm N=171	
**Type of event**	**All AEs**	**Possibly, Probably or Definitely Related Adverse Events[a]**	**All AEs**	**Possibly, Probably or Definitely Related Adverse Events[a]**
Subjects with at least one AE [n (%)]	110(64.7%)	51(30.0%)	104(60.8%)	44(25.7%)
Total # AEs [n (number with grade 3)]	208(1^)	68(0)	239(0)	58(0)
Average # of AEs per subject experiencing AEs.*	1.9	1.3	2.3	1.3
Subjects experiencing a SAE [n (%)]	1^ (0.6)	0(0)	0(0)	0(0)
Total # of SAEs	1	0	0	0

a=relation is to any study intervention. * Calculated as the number of AEs divided by the number of subjects experiencing any AE. ^ Head injury with deep cut secondary to Road traffic accident.

Table S9B. Details of events

	DP arm N=170	Placebo arm N=171
**Adverse event**	**Related events**	**Related events**
Abdominal pains		
Mild	19	9
Moderate	8	13
Dizziness		
Mild	7	4
Moderate	6	9
Diarrhea		
Mild	1	1
Moderate		
Vomiting		
Mild	4	
Moderate	6	3
Tenderness or pain at injection site		
Mild		1
Moderate	1	1
Headache		
Mild	8	8
Moderate	2	4
Fever		
Mild		
Moderate	1	1
Facial puffiness		
Mild		
Moderate	1	
Nausea		
Mild	3	2
Moderate		2
Rapid heart rate		
Mild	1	
Moderate		

Table S10. Impact of dihydroartemisinin piperaquine (DP) versus placebo on vaccine responses, excluding 19 participants in the placebo arm who presented with fever, tested RDT positive for malaria, and were treated with artemether/lumefantrine during the trial

PRIMARY ENDPOINT ANALYSIS				
**Setting**	**n**	**GM(SE)**	**GMR (95% CI)**	**P value**
**BCG-specific IFN-ɣ (8 weeks post-vaccination), SFUs/1 million PBMCs**				
DP	129	294.23 (1.06)	1.03 (0.87-1.22)	0.72
Placebo	116	285.38 (1.06)	Ref.	
**Yellow fever $PRNT_{50}$ titres (4 weeks post-vaccination)**				
DP	146	2021.53 (1.10)	1.17 (0.89-1.53)	0.27
Placebo	126	1733.38 (1.10)	Ref.	
**Yellow fever $PRNT_{90}$ titres (4 weeks post-vaccination)**				
DP	146	183.78 (1.10)	1.22 (0.94-1.58)	0.13
Placebo	126	150.69 (1.09)	Ref.	
***S.* Typhi O:LPS-specific IgG (4 weeks post-vaccination), EU/ml**				
DP	145	300.90 (1.11)	1.14 (0.84-1.55)	0.40
Placebo	126	263.62 (1.12)	Ref.	
**HPV-16-specific IgG (4 weeks post-vaccination), EU/ml***				
DP	83	78.66 (1.19)	0.79 (0.48-1.31)	0.37
Placebo	71	99.26 (1.20)	Ref.	
**HPV-18-specific IgG (4 weeks post-vaccination), EU/ml***				
DP	83	386.50 (1.17)	0.76 (0.49-1.17)	0.22
Placebo	71	505.60 (1.16)	Ref.	
**Tetanus toxoid-specific IgG (24 weeks post-vaccination), IU/ml**				
DP	142	6.17 (1.11)	1.28 (0.96-1.71)	0.091
Placebo	119	4.81 (1.11)	Ref.	
**Diphtheria toxoid-specific IgG (24 weeks post-vaccination), IU/ml**				
DP	142	2.41 (1.06)	0.96 (0.82-1.13)	0.65
Placebo	119	2.50 (1.07)	Ref.	

*Analysis population for HPV vaccine is participants who had not received HPV prior to the trial. SE: standard error; GM: geometric mean; GMR: geometric mean ratio

19 participants who had symptomatic malaria during follow up were excluded from the analysis, all the 19 were in the Placebo arm.

Figure S1. Impact of DP versus placebo on vaccine responses.

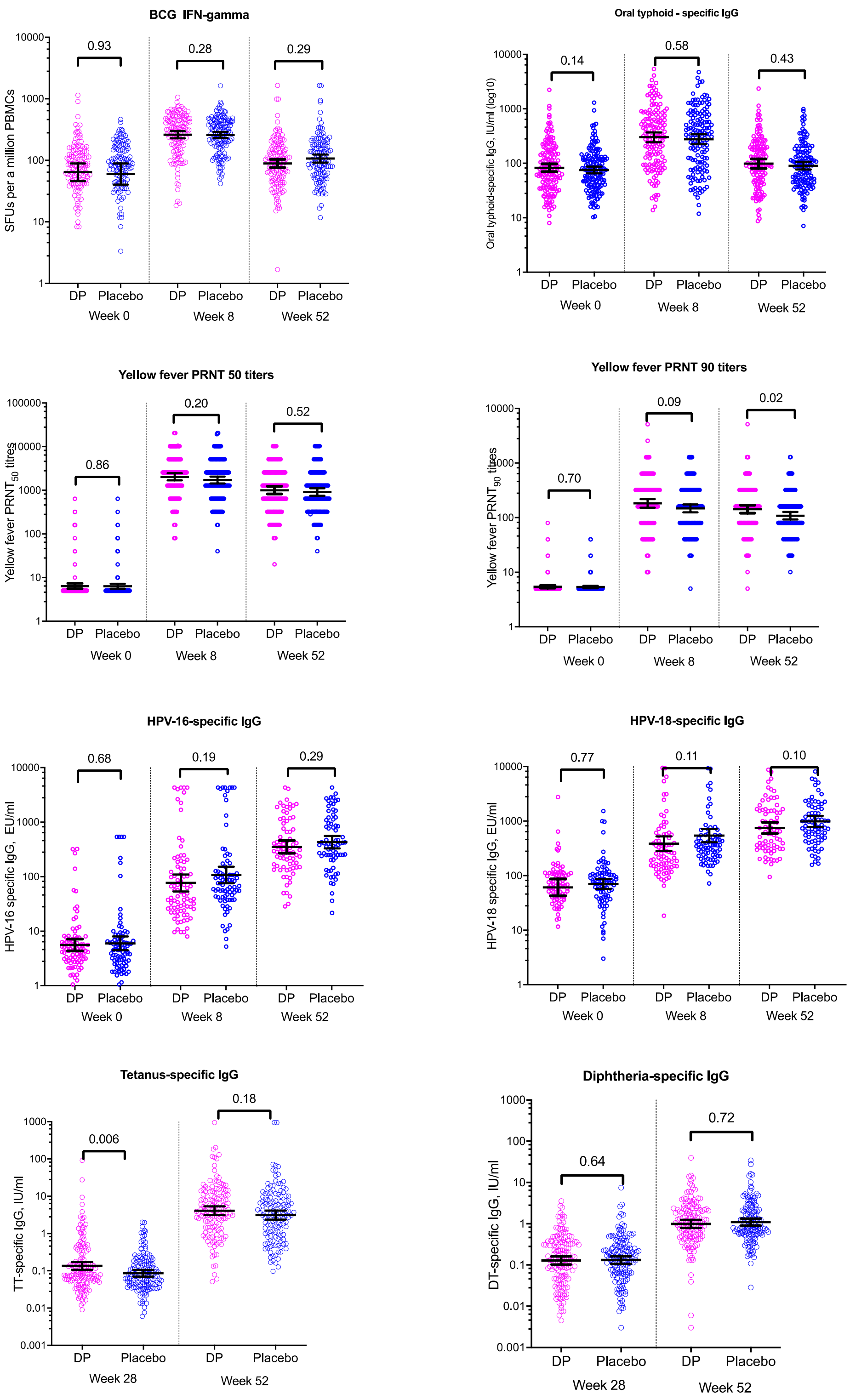

Plots show individual data points, a horizontal line and whiskers denoting the geometric mean and 95% CI, respectively. **SFUs**: ELISpot assay spot forming units; **PBMCs**: peripheral blood mononuclear cells; **PRNT$_{50}$**: plaque reduction neutralizing reference tests at 50% neutralization; **HPV-16**: Human Papillomavirus type 16; **HPV-18**: Human Papillomavirus type 18; **TT**: tetanus toxoid; **DT**: diphtheria toxoid.

Figure S2. Forest plot of comparison of outcomes at the primary outcome time point by trial arm, stratified by sex.

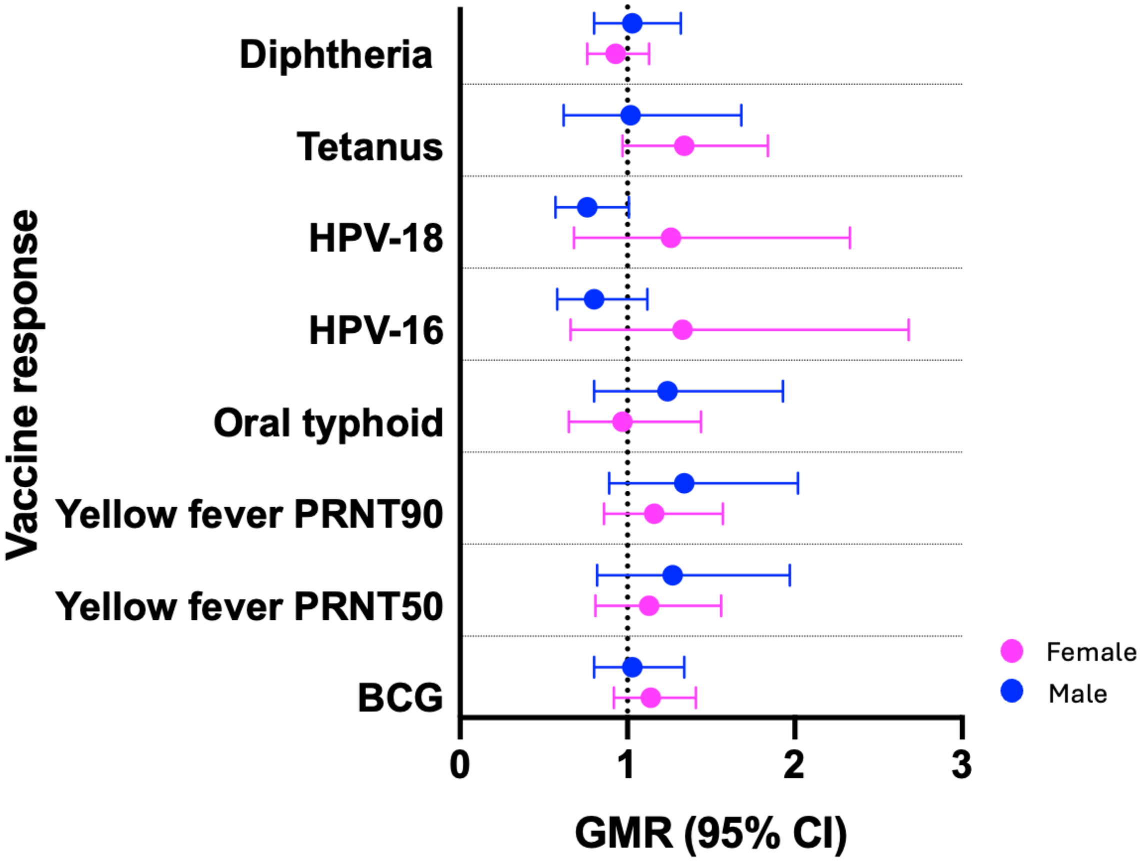

Forest plot shows solid circle denoting the geometric mean and whiskers 95% CIs. **$PRNT_{50}$**: plaque reduction neutralizing reference tests at 50% neutralization; **HPV-16**: Human Papillomavirus type 16; **HPV-18**: Human Papillomavirus type 18.

Figure S3. Malaria infection status by visit, excluding 19 participants in the placebo arm who presented with fever, tested RDT positive for malaria, and were treated with artemether/lumefantrine during the trial

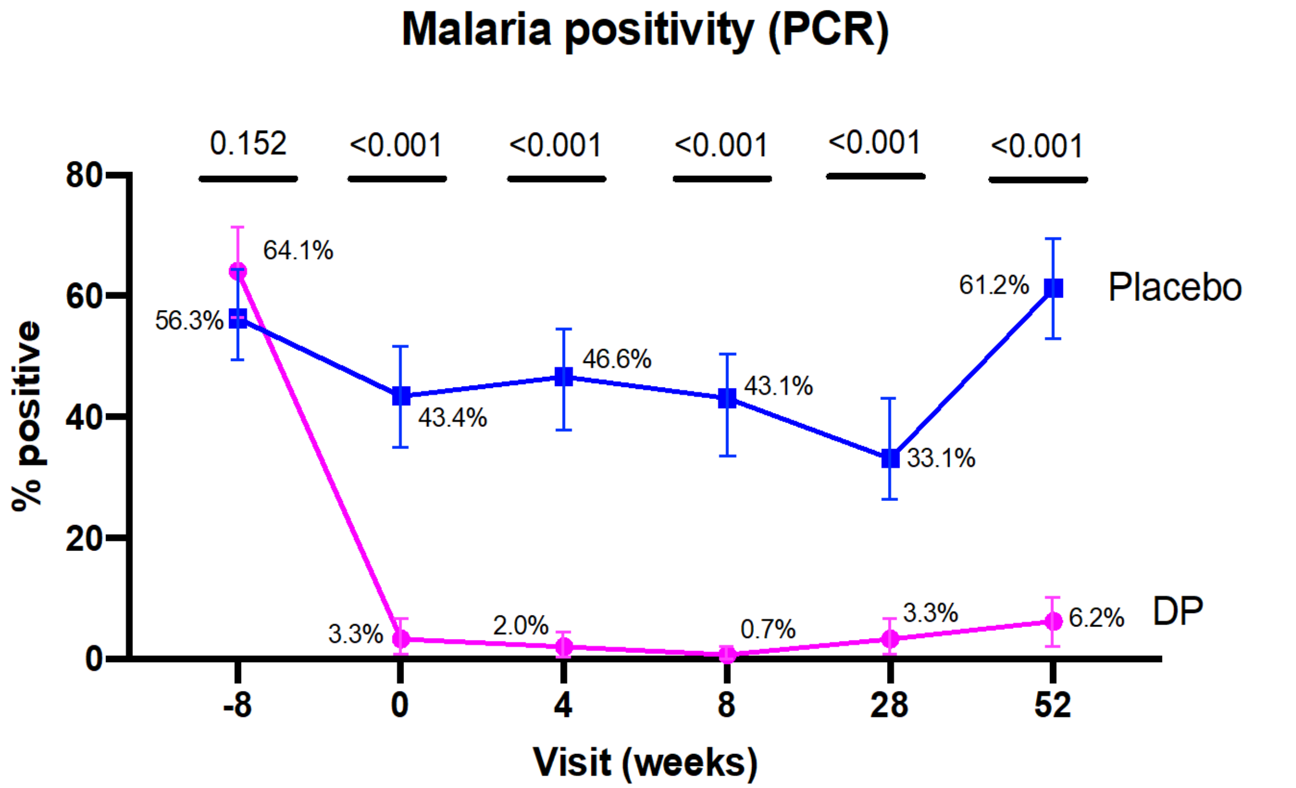

Malaria prevalence at each time point was compared between trial arms using chi-squared tests, respectively. Furthermore, linear mixed models were fitted to compare malaria prevalence ($p<0.0001$) between trial arms, from week -6 to week 52. DP: dihydroartemisinin-piperaquine; PCR: polymerase chain reaction

Supplementary methods

Inclusion and exclusion criteria

Inclusion criteria:

To be included in the trial, participants were required to be attending one of the selected schools in primary 1 to 6, aged 9 to 17 years, willing to provide locator information, and for females, willing to avoid pregnancy during the trial.

Exclusion criteria:

Medical history of serious psychiatric condition, immunodeficiency, endocrine disorder, neurological illness, cancer, cardiovascular, gastrointestinal, liver or renal disease; HIV seropositivity; haemoglobin <82 g/L; positive pregnancy test; currently lactating or intending to become pregnant during the trial period; family history of sudden death attributable to heart condition in a first-degree relative or long QT syndrome; known congenital prolongation of the corrected QT (QTc) interval; history of allergic reaction to immunisation or any allergy to any component of the trial vaccines; tendency to develop keloid scars; moderate or severe acute illness; concurrent oral or systemic steroid medication; concurrent use of other immunosuppressive agents within 2 months prior to enrolment or current use of medications known to prolong the QT interval; use of an investigational medicinal product or non-registered drug, live vaccine, or medical device other than the trial vaccines for 30 days prior to dosing with the trial vaccine, or planned use during the trial period; administration of immunoglobulins and/or any blood products within the three months preceding the planned trial vaccination date.

Strategies to mitigate risk of participant drop out and ensure robust follow-up

To mitigate the risk of participant dropout and ensure robust follow-up, we employed strategies aimed at enhancing participant retention during the study duration. Notably, all immunizations and primary endpoints were strategically scheduled within a single school year, with preparatory meetings conducted in the preceding year. These events were timed to avoid conflicts with holidays and examinations, and close coordination with teachers was maintained to minimize disruptions to school routines.

Detection of *Plasmodium falciparum* DNA by PCR

Prior to the PCR, DNA extraction was performed using the QIAamp DNA Blood Mini Kit (Catalogue number 51106, QIAGEN). Briefly, whole blood pellet samples stored at -80^0C were retrieved and thawed at room temperature. Reagents in the extraction kit were reconstituted as per the manufacturer's instructions. Volumes of retrieved samples were checked and Phosphate Buffered Saline (PBS) was added to samples with inadequate volumes. These were vortexed thoroughly for 2 to 3 minutes. The protease enzyme (20 µl) was pipetted to the bottom of a 2 ml Eppendorf tube, and the whole blood pellet (200 µl) was transferred to the tube. The AL lysis

buffer (200 µl), after thorough mixing, was added and pulse vortexed for 20 seconds. The samples were then placed in a heating block set to 56^{0}C for 15 minutes and thereafter centrifuged at 6000g for 1 minute to ensure no sample was trapped in the Eppendorf tube lid. 200 µl of absolute molecular grade ethanol was added to the sample, pulse vortexed, and then centrifuged. 500 µl of the sample was transferred to a spin column and centrifuged at 6000g for 1 minute, discarding the flow-through thereafter. This process was repeated for the remainder of the sample. The DNA was then purified using consecutive washes with AW1 (500ul, 250ul) and AW2 (500ul) buffers. The QIAGEN AE buffer (150 µl) was used to elute the DNA. The samples were then stored at -20^{0}C until used in the PCR.

The real-time PCR was performed with the ABI 7500 Fast Real-time machine and data processed using 7500 Fast Systems software version 1.5.1. The PCR reaction was performed with a final volume of 25 µl containing 2 µl of DNA 1 µl of PhHV DNA (as an internal control, detailed below) and 22 µl of PCR master mix made of HotStarTaq Master Mix (Catalogue Number 203446), primers, and probes. The primers and probes used in this study are listed below.

PFal-F 5`-CCG ACT AGG TGT TGG ATG AAA GTG TTA A-3`
Plas-171R 5`-AAC CCA AAG ACT TTG ATT TCT CAT AA-3
Pfal114-XS_YY 5`-CTT TCG AGG TGA CTT TTA GAT-3`-BHQ1

Phocine herpes virus (PhHV) DNA, extracted from the Phocine herpes virus (kindly provided by Dr. Martin Schutten, Erasmus Medical Center, Rotterdam, the Netherlands), was included in the PCR master mix, thus distributed to all reaction wells as an internal control to check for PCR inhibition. The PhHV forward primer PhHV-267s (5'-GGGCGAATCACAGATTGAATC-3'), reverse primer PhHV-337as (5'-GCGGTTCCAAACGTACCAA-3') and probe PhHV-305tq (Cy5-5'-TTTTTATGTGTCCGCCACCATCTGGATC-3'-BHQ2) were used for Phocin herpes virus DNA detection. A pool of DNA extracted from *P. falciparum* positive samples was used to set serially diluted standards tested alongside the samples on every plate run. The amplification conditions were 15 minutes at 95°C, 50 cycles of 15 seconds at 95°C, 30s at 60°C, and 30s at 72°C.

***Ex vivo* interferon-γ ELISpot assays to quantify BCG-specific responses**

To quantify BCG-specific responses, we conducted *ex vivo* interferon (IFN)-γ ELISpot assays, using a Human IFN-γ (ALP) ELISpot Flex kit (Mabtech, Sweden) and multiscreen-IP 0.45µm filter 96-well plates (Merck Millipore). ELISpot plates were coated overnight at 4°C with 50 µl of anti-IFN-γ capture antibody (15 µg/ml) dissolved in 0.05 M carbonate-bicarbonate buffer (Sigma Aldrich). The plates were then washed 5 times with sterile 1X PBS (Sigma Aldrich), and blocked (2-5 hours, 37°C) by adding 100 µl/well of R10 medium (10% fetal bovine serum [Sigma Adrich] in RPMI 1640 medium [Thermofisher scientific] supplemented with L-glutamine, streptomycin, HEPES buffer and penicillin [all from Life technologies, UK]). During plate blocking, peripheral blood

mononuclear cells (PBMCs) were isolated from heparinised whole blood by density gradient centrifugation with Histopaque® (Sigma Aldrich). For each study sample, PBMCs (300,000 per test well) were stimulated in duplicate for 18-20 hours at 37°C, 5% CO_2, with BCG (Moscow strain, Serum Institute of India) at a concentration of 200,000 colony forming units per ml, or left unstimulated. Staphylococcal enterotoxin B (SEB; Sigma Aldrich) was used at a final concentration of 10 μg/ml as a positive control, and a 1:1 mix of the 6-kDa early secretory antigenic target and 10-kDa culture filtrate protein (ESAT-6 and CFP-10 recombinant proteins from BEI Resources, USA) used at a final concentration of 2.5 μg/ml for exploratory assessment of tuberculosis infection. Following the 18-20 hour incubation, plates were washed 5 times with 200ul/well PBS containing 0.05% Tween 20 (Sigma Aldrich) and incubated for 2 hours at room temperature with 50 μl per well of a 1/1000 PBS dilution of biotin anti-IFN-γ antibody from the ELISpot kit. After another washing step with PBS-0.05% Tween 20, plates were incubated for 1 hour at room temperature with 50 μl per well of a 1/1000 PBS dilution of a streptavidin-alkaline phosphatase conjugate from the ELISpot kit. Plates were washed, developed for 3-10 minutes with 50 μl per well of 5-Bromo-4-chloro-3-indoxyl phosphate/Nitro blue tetrazolium (BCIP/NBT; Europa Bioproducts), and the reaction stopped by washing the plate under tap water. Plates were allowed to dry in the dark at room temperature and read using an ELISpot reader (Autoimmun Diagnostika Gmbh iSpot , Strassberg, Germany) running AID ELISpot software v.7.0. Spot-forming units (SFUs) per well were manually verified to remove artefacts.

We performed QC through a number of steps: 1) for each sample, we checked to ascertain whether the PBMC isolation procedure was conducted within eight hours after sample collection; 2) we inspected each ELISpot plate visually for quality and completeness of labelling (sample IDs, date, time point and antigens) and compared the plate picture to the exported spot count spreadsheet to ensure the correct data had been exported; 3) we checked the calculated data in the exported database to ensure the background subtraction, average of duplicate wells and multiplication up to spot forming units per million PBMC had been performed correctly; 4) we assessed whether the unstimulated well and SEB well controls for each assay were within the accepted range.

Results were reported as SFUs per a million PBMCs, calculated sequentially by 1) subtracting mean SFUs of unstimulated wells from mean SFUs of duplicate antigen wells, and 2) correcting for the number of PBMCs per well (300,000). Samples that had more than 83.3 SFUs per a million PBMCs in the unstimulated well were considered invalid and not included in the final analysis.

Yellow Fever plaque reduction neutralizing reference test (PRNT)

A plaque reduction assay as described by Beaty *et al.*[1] was used. Briefly, Vero cells at a concentration of 65,000 cells/ml were seeded into 6-well plates (Greiner Bio-One GmbH, Germany) at a volume of 3 ml/well. Cells were

cultured in growth medium (1X Eagle's Minimum Essential Medium, 8% heat inactivated fetal bovine serum, 100 units penicillin/streptomycin, gentamycin 50 mg/ml and fungizone 1 mg/ml) at 37°C (with 5% CO_2) for 3–4 days. Culture medium was then removed from the cell monolayer by dumping. Test plasma were inactivated at 56°C for 30 min to remove complement factor, serially (two-fold) diluted from 1:10 to 1:20480 in BA-1 diluent (10X M199 Hanks' Salts without L-Glutamine, 5% Bovine Serum Albumin, 1M TRIS-HCL pH 7.5, L-Glutamine, sodium bicarbonate 7.5%, 100X penicillin/streptomycin, 1000X fungizone in sterile water), and mixed with approximately 200 Plaque Forming Units (PFU) of a reference YF-17D virus preparation. The plasma-virus mixture (0.1 ml) was added to the confluent monolayer of Vero cells in each well and incubated at 37°C (with 5% CO_2) for 1 hour. The first overlay medium (comprising Miller's 2X Yeast Extract-Lactalbumin hydrolysate medium, 10X Earle's Buffered Salts Solutions, 2% fetal bovine serum, 1000X fungizone, 1000X Gentamycin, and 2% low-melting agarose) was added, 3 ml per well, and allowed to solidify for 30 minutes at room temperature. The plates were incubated at 37°C with 5% CO_2 for 4 days. To stain cell layers, a Neutral Red dye (Sigma Aldrich) second overlay was added, 2 ml per well, and allowed to solidify for 30 minutes at room temperature. After this second overlay, plates were incubated at 37°C in 5% CO_2 for 2 days: plaques were counted first on day 1 and the final score documented on day 2 to establish 50% and 90% neutralization titres. Back titration plates were established to ensure infectivity of cell monolayer and standardization of virus to 200 PFU/0.1 ml. Neutralisation titres <1:10 were considered negative. Titres of 1:10 were interpreted as borderline. The PRNT antibody titres presented refer to the reciprocal of the last plasma dilution that reduced by 50% (PRNT50) or 90% (PRNT90) the number of virus plaque clusters infected by 100 PFU/0.1 ml of the reference 17D virus preparation.

For quality control, we used a high titre positive control (PC), with the last six titre dilution range, from 640 to 20480. So long as the PC titre was within the expected range and did not vary by greater than 4-fold, the assay passed quality control. Furthermore, we ran back-titrations of the virus inoculum (standardised to 200 PFU/0.1 ml) to determine the end-point specimen antibody titre at 50% or 90% neutralisation. The number of virus plaques infected at 50% neutralisation and at 90% neutralisation were expected to be within an approximate range of 25-100 and 5-20, respectively.

Detection of plasma IgG against *Salmonella* Typhi O-lipopolysaccharide (O:LPS)

Specific IgG to *S. Typhi* O-lipopolysaccharide (O:LPS) was measured by an in-house ELISA. Nunc Maxisorp 96-well plates (Thermo Fisher) were coated overnight at 4ºC with 50µl/well of 10 µg/ml of *S.* Typhi O:LPS (Sigma L2387) in bicarbonate (Na_2CO_3 + $NaHCO_3$) buffer (0.1M, pH 9.6). Plates were washed with phosphate-buffered saline (PBS 1X)-Tween 20 (0.05%) solution, blocked with 200µl of 5% skimmed milk diluted in PBS-Tween 20 for 1 hour at room temperature (RT), washed again and incubated for 2 hours at RT with 50µl of test plasma

samples (diluted 1/320 with 1% skimmed milk in PBS-Tween 20) and two-fold serially diluted standard sera (top concentration 20 EU/ml). Standards were derived from a pooled sample generated from sera of known O:LPS-specific IgG titres, kindly provided by the Oxford Vaccine Centre Biobank. These sera had been collected from the highest responders to O-antigen following challenge with *S.* Typhi in a controlled human infection study.[2] Following test and standard sample incubation, plates were washed and O:LPS-specific IgG binding detected by incubating the plates for 1 hour at RT with goat anti-human IgG-horseradish peroxidase conjugate (Insight Biotechnology, UK), diluted 1/6000 in 1% skimmed milk–PBS-Tween 20. Plates were washed and developed by addition of 100µl of o-phenylenediamine (Sigma-Aldrich) and reactions stopped after 5 minutes with 30µl of 2M Sulphuric acid. Optical density (OD) values were measured at 490nm (reference wavelength 630nm) on a 96-well plate ELISA reader (BioTek ELx808, USA). Nominal ELISA units (EU/ml), representing O:LPS-specific IgG titres, were interpolated from standard curves using a five-parameter curve fit using Gen5 data collection and analysis software (BioTek Instruments Inc, Vermont, Winooski, USA).

Detection of plasma IgG against Human Papillomavirus type 16 (HPV-16) and HPV-18

Anti-HPV-16 and HPV-18 IgG concentrations were measured by ELISA, as previously described.[3-6] Nunc Maxisorp 96-well plates (Thermo Fisher) were coated with 100 µl of HPV-16 L1 virus-like particles (VLP) at a concentration of 2.7 µg/ml, or with HPV-18 L1-L2 VLP at a concentration of 2 µg/ml and incubated at 4°C overnight. Plates were washed three times with a 1X phosphate-buffered saline (PBS)-Tween 20 (0.25%) solution, and blocked for 90 minutes at room temperature (RT) with 4% skimmed milk diluted in a 1X PBS-0.25% Tween 20 solution. The plates were washed three times and and incubated (with gentle shaking) for 1 hour at RT with 100 µl of test plasma samples, assay controls and two-fold serially diluted standard sera. Pre-vaccination test plasma samples were diluted 1/50 (HPV-16 assay) or 1/200 (HPV-18 assay) with blocking buffer, while post-vaccination plasma samples were diluted 1/400 for both HPV-16 and HPV-18 assays. Standard sera were used at a top concentration of 1.28 EU/ml and 8.2425 EU/ml for HPV-16 and HPV-18 assays, respectively. Following test and standard sample incubation, plates were washed four times and further incubated for 1 hour at RT with peroxidase-labeled goat anti-human IgG (KPL, Gaithersburg, Maryland). Plates were then developed with a tetramethylbenzidine substrate solution (KPL, Inc.) for 25 minutes in the dark at room temperature. Next, the reaction was stopped by adding 100 µl of 0.36N H_2SO_4 to each well. Optical density (OD) values were measured at 450 nm (reference wavelength 630 nm) on a 96-well plate ELISA reader (BioTek ELx808, USA). Nominal ELISA units (EU/ml), representing HPV-16 L1 VLP- and HPV-18 L1-L2 VLP-specific IgG titres, were interpolated from standard curves using a five-parameter curve fit using Gen5 data collection and analysis software (BioTek Instruments Inc, Vermont, Winooski, USA).

For quality control (QC), the acceptable R^2 for the standard curve was ≥ 0.990 and the average optical density (OD) range of the top standard was 2.0-4.0. The acceptable OD of the last (8th) standard dilution was ≤0.25. The percentage difference in ODs between standard dilutions (i.e. n and n+1 dilution) was expected to be ≥ 0.3. Plates were repeated if they failed to meet these standard curve criteria. The calculated negative control cut-off was 4±3 EU/ml and 60±10 EU/ml for HPV-16 and HPV-18, respectively. Plates whose negative control concentration was above the cut-offs were repeated. The calculated positive control concentration was 450±10 EU/ml and 4500±10 EU/ml for HPV-16 and HPV-18 respectively. Background signal was measured by a blank whose OD was expected to be ≤0.05. Higher ODs indicated assay contamination and plates were repeated.

ELISA measurement of anti-diphtheria and anti-tetanus IgG

Nunc Maxisorp 96-well plates (Thermo Fisher) were coated with 50 µl of either 2 Lf units per ml of diphtheria toxoid (NIBSC product code 13/212) per ml or 0.56 Lf units per ml of tetanus toxoid (NIBSC product code 02/232) in Na_2CO_3/$NaHCO_3$ buffer (0.1M, pH 9.6) overnight at 4°C. Plates were washed with 0.05% Tween 20 in 1X phosphate-buffered saline (PBST) and blocked for 1 hour with 5% skimmed milk powder in PBST at 37 °C. The plates were washed four times and and incubated for 2 hours at 37 °C with 50 µl of test plasma samples, and serially diluted WHO International Standard anti-toxins for diphtheria (NIBSC 10/262) or tetanus (NIBSC 13/240). Samples and standards were prepared in PBST + 1% skimmed milk (assay buffer). Pre-vaccination test samples were added at a dilution of 1/150, while post-vaccination plasma samples were added at a dilution of 1/300 in assay buffer. The standards were used at a top concentration of 3 IU/ml and 0.125 IU/ml for the tetanus and diphtheria assays, respectively. Plates were washed four times and incubated for 1 hour at 37 °C with 50 µl of polyclonal rabbit anti-human IgG HRP-conjugate (Agilent Dako, CA, USA) diluted 1/3000 in assay buffer. After another washing step, plates developed by adding 100 µl/well of o-phenylenediamine (Sigma-Aldrich) and reactions stopped after 5 minutes with 25 µl/well of 2M sulphuric acid. Optical density (OD) values were measured at 490nm (reference wavelength 630nm) on a 96-well plate ELISA reader (BioTek ELx808, USA). Tetanus and diphtheria toxoid-specific IgG concentrations (IU/ml) were interpolated from standard curves using a five-parameter curve fit using Gen5 data collection and analysis software (BioTek Instruments Inc, Vermont, Winooski, USA).

Plasma detection of *Schistosoma* circulating anodic antigen

Plasma samples collected at screening, week 0, week 8, week 28 and week 52 were retrospectively analysed for *Schistosoma* circulating anodic antigen (CAA) using the up-converting phosphor lateral flow (UCP-LF) assay with

a SCAA20 test format, with a 30 pg/ml positivity threshold.[7] This limit of detection was previously determined by spiking CAA (standard series) in negative serum from non-endemic healthy individuals and analyzed against a large set of confirmed CAA negative controls from different endemic regions. Based on this, quality control was conducted on test materials provided for the study to guarantee a lower limit of detection of 30 pg/ml. All samples above this level (≥30 pg/ml) are regarded as CAA-positive.

For the current study, human negative serum (obtained from the Uganda blood bank, Nakasero) was spiked with a known concentration of CAA standard (100,000 pg/ml) and diluted up to eight standard points, with two negative controls. These were used to generate a standard curve to quantify the plasma sample CAA levels. Previous work has shown that there is no difference between standard curves with spiked CAA in serum and in plasma.[7] Therefore, plasma and standards (50 µl) were extracted with an equal volume of 4% w/v trichloroacetic acid (TCA; Merck Life Science NV, the Netherlands), vortexed and incubated at ambient temperature for five minutes. Thereafter, samples and standards were briefly vortexed and spun at 13,000 g for five minutes. The resulting supernatant (20µl) was added to the wells containing 100 ng dry UCP particles[8] (400 nm Y2O2S:Yb3+, Er3+) coated with mouse monoclonal anti-CAA antibodies[9] hydrated with 100µl of high salt lateral flow buffer (HSLF: 200 mM Tris pH8, 270 mM NaCl, 0.5% (v/v) Tween-20, 1% (w/v) BSA). These were incubated for one hour at 37°C while shaking at 900 rpm. The CAA lateral flow strips[10] were labeled with the standard and sample identifications and then placed in the wells on the UCP plate. The samples and standards were allowed to flow and left to dry overnight. The strips were then analysed using the Labrox Upcon reader (Labrox Oy, Turku, Finland). The test line signals (T; relative fluorescent units, peak area) were normalized to the flow control signals (FC) of the individual strips and the results were expressed as ratio values.

Stool PCR detection of *Schistosoma mansoni*, *Necator americanus* and *Strongyloides stercoralis* DNA

Stool samples collected at the screening timepoint (week -6) were retrospectively analysed for *helminth* DNA by PCR.

Stool samples stored at -80^{0}C in 95% molecular grade ethanol were retrieved and thawed at room temperature. Total DNA (and hence helminth DNA, if present) was extracted from the stool samples using the Fast DNA Spin Kit for Feces (catalogue number 116570200 , MP Biomedicals Germany GmbH) to determine (using multiplex real-time PCR) *S. mansoni, Strongyloides stercoralis* and hookworm (*Necator americanus*) infections.

The DNA extraction procedure was conducted with minor changes to the manufacturer's instructions as follows: samples were left to thaw at room temperature (RT) and then vortexed for five seconds to homogenise the ethanol-stool mixture. The homogenized mixture (0.5 ml) was transferred into a safe-lock microcentrifuge (Eppendorf®) tube and centrifuged at 13000 rpm for 3 minutes to get rid of the ethanol. The pellet was re-suspended in a 2ml lysing matrix E tube, containing 825 µl of sodium phosphate buffer and 275 µl of PLS buffer.

For solid samples, 300-500mg of stool were transferred to the lysis tube using a sterile loop. The samples were centrifuged for 5 minutes at 14000 g and the supernatant was decanted. 978 µl Sodium Phosphate Buffer and 122 µL MT Buffer were added to the lysing matrix E tube and vortexed briefly to mix the contents. The samples were then homogenised in the FastPrep® 24 instrument (MP Biomedicals Germany GmbH) at setting 6.0 m/s for 40 seconds and thereafter centrifuged at 14000 g for 5 minutes. The supernatant was then transferred to a clean 2.0 ml centrifuge tube and 250 µl of PPS solution was added. The samples were then shaken vigorously to mix, incubated at 4°C for 10 minutes, and centrifuged at 14000g for 2 minutes. During the centrifugation step, we added 1 ml of binding matrix solution to a clean 15 ml conical tube. The supernatant was then transferred to the 15 ml tube containing the binding matrix. These were mixed gently by hand for 3 to 5 minutes. The samples were then centrifuged at 14000 g for 2 minutes and the supernatant discarded. The binding mixture pellet was then washed by gently re-suspending it in 1 mL Wash Buffer #1. Two spins were performed by first transferring 600 µL of the binding mixture to a filter tube and centrifuged at 14000 g for 1 minute. The catch tube was then emptied and the remaining binding mixture was added to the filter tube and centrifuged as before. The catch tube was emptied again and 500 µL of prepared Wash Buffer #2 (concentrated salt wash solution, reconstituted with absolute molecular grade ethanol) was added to the filter tube and re-suspended by gently pipetting up and down to dislodge the pellet. The samples were centrifuged at the same speed for 2 minutes and the flow-through discarded. The samples were centrifuged again for 2 minutes to extract residual ethanol from the binding matrix and dry the sample. The filter bucket was transferred to a clean 1.9 mL Catch Tube and 100 µL TES elution buffer added. The tube was stirred with a pipette tip to resuspend the pellet. The samples were then centrifuged for 2 minutes to elute purified DNA into the clean catch tube. The samples were stored at -20°C until used for PCR (below).

The multiplex real-time PCR was adapted from existing procedures.[11,12] Below are the specific forward (F) and reverse (R) primers and TaqMan® probes that were used to simultaneously detect DNA from three helminth species:

Necator americanus

Na58F: 5’-CTGTTTGTCGAACGGTACTTGC-3’

Na158R: 5’- ATAACAGCGTGCACATGTTGC-3’

Nec-2-FAM (MGB): FAM-5’-CTGTACTACGCATTGTATAC-3’-XS

Nec-3-FAM (MGB): 5’-CTGTACTACGCATTGTATGT-3’

Schistosoma mansoni

Ssp48F: 5’-GGTCTAGATGACTTGATYGAGATGCT-3’

Ssp124R: 5’-TCCCGAGCGYGTATAATGTCATTA-3’

Ssp78T-RT: Texas Red-5’-TGGGTTGTGCTCGAGTCGTGGC-3’-BHQ2

Strongyloides stercoralis

Stro18S-1530F: 5’-GAATTCCAAGTAAACGTAAGTCATTAGC-3’

Stro18S-1630R: 5’-TGCCTCTGGATATTGCTCAGTTC-3’

Stro-4-TRBhq2-VIC- 5’-ACACACCGSCCGTCGCTGC-3’

Phocine herpes virus (PhHV) DNA, extracted from the Phocine herpes virus (kindly provided by Dr. Martin Schutten, Erasmus Medical Center, Rotterdam, the Netherlands), was included in the PCR master mix, thus distributed to all reaction wells as an internal control to check for PCR inhibition. The PhHV forward primer PhHV-267s (5’-GGGCGAATCACAGATTGAATC-3’), reverse primer PhHV-337as (5’-GCGGTTCCAAACGTACCAA-3’) and probe PhHV-305tq (Cy5-5’ TTTTTATGTGTCCGCCACCATCTGGATC-3’-BHQ2) were used for Phocin herpes virus DNA detection. A positive pool was included on the plate for every run as a test control. The positive pool was made up of a mixture of DNA from samples (from among the study samples) that were highly positive for *S. mansoni* and *N. americanus* on Kato-Katz, and for *S. stercoralis* by PCR (conducted on samples from a previous study). The amplification conditions were 15 minutes at 95°C, 50 cycles of 15 seconds at 95°C, 30s at 60°C and 30s at 72°C. DNA amplification, detection and data analysis were attained with the ABI 7500 Fast Real time machine and 7500 Fast systems software version 1.5.1.

Key search terms

The search terms were "WHO-licenced vaccines" AND ("malaria infection" OR "malaria treatment") AND "immune responses"

Actual keywords:

(("immune response" OR immunity OR immunomodulation OR "cell-mediated immune response" OR "humoral immune response" OR "antibody response" OR "immune-reaction" OR "immunologic response" OR produc* "NEAR/3" (antibod* OR cytokine OR response*) OR (vaccin* OR immun* OR antibod* OR cytokine) "NEAR/3" response* OR antigenicity OR immunogenicity))

AND

(malaria OR "malaria, avian" OR "malaria, falciparum" OR "malaria, vivax" OR "malaria, chabaudi" OR plasmodium))

AND

(cholera OR dengue OR diphtheria OR "hepatitis a" OR "hepatitis b" OR hbv OR "hepatitis e" OR "haemophilus influenzae" OR influenza OR "human papilloma virus" OR hpv OR "japanese encephalitis" OR measles OR meningococcus OR meningococcal OR mumps OR pertussis OR pneumococcus OR pneumococcal OR poliomyelitis OR polio OR rabies OR rotavirus OR rubella OR tetanus OR "tick-borne encephalitis" OR tuberculosis OR typhoid OR varicella OR "yellow fever" OR "covid-19" OR ebola OR "bcg vaccine" OR bcg OR "bacille calmette-guerin")

AND

(vaccines OR vaccines OR "viral vaccines" OR immunization OR "immunization schedule" OR "immunization, secondary" OR vaccination OR "mass vaccination" OR "immunization programs" OR (vaccin* OR immuniz* OR immunis*))

References

1. Beaty BJ, Calisher CH, Shope RE. Arboviruses. In: Schmidt NJ, Lennette DA, Lennette ET, Lennette EH, Emmons RW, eds. Diagnostic Procedures for Viral, Rickettsial and Chlamydial Infections. 7th ed. Washington DC: American Public Health Association; 1995: 204-5.
2. Gibani MM, Jin C, Shrestha S, et al. Homologous and heterologous re-challenge with Salmonella typhi and Salmonella paratyphi A in a randomised controlled human infection model. *PLoS neglected tropical diseases* 2020; **14**(10): e0008783.
3. Dauner JG, Pan Y, Hildesheim A, Kemp TJ, Porras C, Pinto LA. Development and application of a GuHCl-modified ELISA to measure the avidity of anti-HPV L1 VLP antibodies in vaccinated individuals. *Mol Cell Probes* 2012; **26**(2): 73-80.
4. Dauner JG, Pan Y, Hildesheim A, Harro C, Pinto LA. Characterization of the HPV-specific memory B cell and systemic antibody responses in women receiving an unadjuvanted HPV16 L1 VLP vaccine. *Vaccine* 2010; **28**(33): 5407-13.
5. Pinto LA, Kemp TJ, Torres BN, et al. Quadrivalent Human Papillomavirus (HPV) Vaccine Induces HPV-Specific Antibodies in the Oral Cavity: Results From the Mid-Adult Male Vaccine Trial. *The Journal of infectious diseases* 2016; **214**(8): 1276-83.
6. Miller CN, Kemp TJ, Abrahamsen M, et al. Increases in HPV-16/18 antibody avidity and HPV-specific memory B-cell response in mid-adult aged men post-dose three of the quadrivalent HPV vaccine. *Vaccine* 2021; **39**(37): 5295-301.
7. Corstjens PL, De Dood CJ, Kornelis D, et al. Tools for diagnosis, monitoring and screening of Schistosoma infections utilizing lateral-flow based assays and upconverting phosphor labels. *Parasitology* 2014; **141**(14): 1841-55.
8. Corstjens PL, Li S, Zuiderwijk M, et al. Infrared up-converting phosphors for bioassays. *IEE Proc Nanobiotechnol* 2005; **152**(2): 64-72.
9. Corstjens PL, van Lieshout L, Zuiderwijk M, et al. Up-converting phosphor technology-based lateral flow assay for detection of Schistosoma circulating anodic antigen in serum. *Journal of clinical microbiology* 2008; **46**(1): 171-6.
10. Corstjens P, Hoekstra PT, de Dood CJ, van Dam GJ. Utilizing the ultrasensitive Schistosoma up-converting phosphor lateral flow circulating anodic antigen (UCP-LF CAA) assay for sample pooling-strategies. *Infect Dis Poverty* 2017; **6**(1): 155.

11. Verweij JJ, Brienen EA, Ziem J, Yelifari L, Polderman AM, Van Lieshout L. Simultaneous detection and quantification of Ancylostoma duodenale, Necator americanus, and Oesophagostomum bifurcum in fecal samples using multiplex real-time PCR. *The American journal of tropical medicine and hygiene* 2007; **77**(4): 685-90.
12. Verweij JJ, Canales M, Polman K, et al. Molecular diagnosis of Strongyloides stercoralis in faecal samples using real-time PCR. *Transactions of the Royal Society of Tropical Medicine and Hygiene* 2009; **103**(4): 342-6.
