# Supplementary material for: The effect of intermittent preventive treatment for malaria with dihydroartemisinin–piperaquine on vaccine-specific responses among schoolchildren in rural Uganda (POPVAC B): a double-blind, randomised controlled trial
Source: Lancet Glob Health. 2024 Oct 16;12(11):e1838–48. doi: 10.1016/S2214-109X(24)00281-X (PMC11483247; doi:10.1016/S2214-109X(24)00281-X)
Supplement: Equitable Partnership Declaration [file mmc3.pdf]

THE LANCET
Global Health

Supplementary appendix 3

This Equitable Partnership Declaration (EPD) was submitted by the authors, and we reproduce it as supplied. It has not been peer reviewed. *The Lancet*'s editorial processes have not been applied to the EPD.

Supplement to: Zirimenya L, Natukunda A, Nassuuna J, et al. The effect of intermittent preventive treatment for malaria with dihydroartemisinin–piperaquine on vaccine-specific responses among schoolchildren in rural Uganda (POPVAC B): a double-blind, randomised controlled trial. *Lancet Glob Health* 2024; **12:** e1838–48.

Equitable Partnership Declaration questions

Researcher considerations

1. Please detail the involvement that researchers who are based in the region(s) of study had during a) study design; b) clinical study processes, such as processing blood samples, prescribing medication, or patient recruitment; c) data interpretation; and d) manuscript preparation, commenting on all aspects. If they were not involved in any of these aspects, please explain why.

   This question is intended for international partnerships; if all your authors are based in the area of study, this question is not applicable.

   This should include a thorough description of their leadership role(s) in the study. Are local researchers named in the author list or the acknowledgements, or are they not mentioned at all (and, if not, why)? Please also describe the involvement of early career researchers based in the location of the study. Some of this information might be repeated from the Contributors section in the manuscript. Note: we adhere to ICMJE authorship criteria when deciding who should be named on a paper.

**a) Study design:**<br><br>AME, ELW, GN, SC and PK conceptualised the trial. All, except ELW, are based in Uganda, the region of study.
**b) Clinical study processes:**<br><br>These were conducted by researchers based in the region of study. These researchers are either listed as authors under the article title or as members of the POPVAC trial team, whose names are listed at the end of the article. LZ was the trial project leader in charge of clinical study processes; LZ, AW, CZ, CN, EN, FA, MS and SK conducted field and clinical work; GN led the laboratory work and JN, BW, JKabagenyi, JKayiwa, PNK performed the laboratory assays.<br><br>POPVAC trial team members not listed as individually named authors under the article title participated in field work and administration, data management, laboratory testing, quality control, care of participants and administration of trial interventions.
**c) Data interpretation:**<br><br>AN (based in Uganda) curated the data, and undertook the formal analyses, in collaboration with ELW (based in the United Kingdom).
**d) Manuscript preparation:**

GN, JN, AN, LZ, ELW and AME wrote the original draft. All, except ELW, are based in Uganda, the region of study. All authors reviewed and edited the manuscript.

2. Were the data used in your study collected by authors named on the paper, or have they been extracted from a source such as a national survey? ie, is this a secondary analysis of data that were not collected by the authors of this paper. If the authors of this paper were not involved in data collection, how were data interpreted with sufficient contextual knowledge?

   The Lancet Global Health *believe contextual understanding is crucial for informed data analysis and interpretation.*

   The data used in our study were collected by authors named on the paper.

3. How was funding used to remunerate and enhance the skills of researchers and institutions based in the area(s) of study? And how was funding used to improve research infrastructure in the area of study?

   Potentially effective investments into long-term skills and opportunities within institutions could include training or mentorship in analytical techniques and manuscript writing, opportunities to lead all or specific aspects of the study, financial remuneration rather than requiring volunteers, and other professional development and educational opportunities.

   Improvements to research infrastructure could be funding of extended trial designs (such as platform trials) and use of master protocols to enable these designs, establishment of long-term contracts for research staff, building research facilities, and local control of funding allocation.

   Skills:
   Researchers (based in the study region) who conducted the study, and who were/ are staff of the MRC/UVRI and LSHTM Uganda Research Unit, had part or all of their official employment during the study facilitated by funding obtained to run the POPVAC trials. Researchers not officially employed by the MRC/UVRI and LSHTM Uganda Research Unit (such as members of Village Health Teams) received approved allowances commensurate with their involvement in the study.

   Some of the researchers leading the work utilised available funding to enhance their skills; for example, by attending conferences, laboratory training (for example, in conducting the *Schistosoma mansoni* CAA assay), and attending short courses in areas such as vaccinology and

manuscript writing. Moreover, some researchers utilised the rich sample archive provided by the POPVAC trials to generate new research ideas leading to independent funding.

Research infrastructure:

The infrastructure required for this work was largely provided by the MRC/UVRI and LSHTM Uganda Research Unit. The funding provided for the project contributed to maintenance and overhead costs. The allocation of funds was managed by the in-country team. Key to this were laboratory facilities, well equipped to process and analyse biological samples essential for assessing the study endpoints. Furthermore, the organization ensured the integrity of investigational products by furnishing robust storage facilities for vaccines and other study supplies, meticulously maintained to adhere to specified temperature and storage conditions. In addition, a data management system, tailored to the unique requirements of the trial, was meticulously implemented to collect, store, and analyse study data accurately, ensuring compliance with regulatory requirements and safeguarding participant confidentiality. With further support from the MRC/UVRI and LSHTM Uganda Research Unit, meticulous quality assurance and monitoring mechanisms were diligently established to ensure strict adherence to the study protocol, regulatory guidelines, and Good Clinical Practice standards.

4. How did you safeguard the researchers who implemented the study?

Please describe how you guaranteed safe working conditions for study staff, including provision of appropriate personal protective equipment, protection from violence, and prevention of overworking.

We prioritised availability and ensured use of appropriate personal protective equipment tailored to the specific risks associated with each aspect of the study. Examples included gloves, visors, shoes and laboratory/clinical coats in the labs and in clinic settings; protective clothing and safe driving training for motorbike riders. As part of its travel policy, the MRC/UVRI and LSHTM Uganda Research Unit ensured staff transportation for fieldwork activities. Overall, in line with policy of the MRC/UVRI and LSHTM Uganda Research Unit, we instituted stringent safety protocols and

guidelines informed by best practices and regulatory standards. These protocols encompassed procedures for handling potentially hazardous materials and conducting risk assessments.
To address the risk of violence in field settings where researchers might encounter unfamiliar or potentially volatile environments, we implemented robust security measures. These included thorough site visits prior to fieldwork, accompanied visits in certain contexts, and communication protocols to ensure researchers could promptly alert appropriate authorities or support personnel if they felt unsafe. To prevent overworking, we rotated staff between the field and the mainland campus in Entebbe.

Benefits to the communities and regions of study

5. How does the study address the research and policy priorities of its location?

How were the local priorities determined and then used to inform the research question? Who decided which priorities to take forward? Which elements of the study address those priorities?

Plans for the study were developed by the Uganda team, with input from colleagues at the Vector Control Division and Expanded Programme on Immunisation at the Uganda Ministry of Health. Additionally, the concepts involved were discussed with colleagues at Jinja District Council and with community leaders and Village Health Teams from Buwenge subcounty. In addition to further discussions with these stakeholders, we engaged teachers and parents in planning the detailed standard operating procedures for the study. Prior to recruitment we held meetings to discuss the proposed work with teachers, parents and pupils and to address their questions. As well, meetings with village leaders and village members were held to discuss the study and announcements were made in villages regarding the study's start. At the end of the study, results were shared with these stakeholders.

6. How will research products be shared in the community of study?

For instance, will you be providing written or oral layperson summaries for non-academic information sharing? Will study data be made available to institutions in the region(s) of study? The Lancet Global Health *encourages authors to translate the summary (abstract) into relevant languages after paper editing; do you intend to translate your summary?*

Results from this study have been shared with the local study communities. We held meetings with research participants, schools and community leaders in the region of the study, to share the results. The results were also shared with public health policy makers or their representatives:

these included collaborators from the Uganda Ministry of Health, who were members of the trial steering group. We also participated in meetings with leaders both at county and district level. We provided simple practical results digests and briefs, highlighting the extent to which our findings have direct policy implications. For example, results have been presented at a science summit held by the Ministry of Health National Tuberculosis and Leprosy Control Programme.

Institutions in Uganda, and beyond, will have the opportunity to access the study data. The de-identified individual participant data that underlie the results reported in this article are stored in a non-publicly available repository (LSHTM Data Compass), together with a data dictionary. Data are available on request via https://doi.org/10.17037/DATA.00003759. Researchers who would like to access the data may submit a request through LSHTM Data Compass, detailing the data requested, the intended use for the data, and evidence of relevant experience and other information to support the request. The request will be reviewed by the Principal Investigator in consultation with the MRC/UVRI and LSHTM data management committee, with oversight from the UVRI and LSHTM ethics committees. In line with the MRC policy on Data Sharing, there will have to be a good reason for turning down a request. Patient Information Sheets and consent forms specifically referenced making anonymised data available and this has been approved by the relevant ethics committees. Researchers given access to the data will sign data sharing agreements which will restrict the use to answering pre-specified research questions.

We have translated the abstract of the paper into the Luganda language. This translation will be accessible with the published paper.

7. How were individuals, communities, and environments protected from harm?

a) *How did you ensure that sensitive patient data was handled safely and respectfully? Was there any potential for stigma or discrimination against participants arising from any of the procedures or outcomes of the study?*

Personal data were handled in compliance with GDPR. In the datasets individuals are identified only by a number. Paper documents that would allow names to be linked to the number are kept securely. We performed HIV testing prior to enrolment into the study that involved pre-test and post-test counselling. If a participant was confirmed positive, referral to an HIV care provider was done. For female participants, we conducted pregnancy testing which was done at baseline and before immunisation on each immunisation day. When confirmed positive, counselling was done, and referral for antenatal care done. At baseline, these participants were excluded whereas

during follow up, participants received no further trial-related interventions and were followed up until after delivery to ensure that both mother and baby were well.

b) *Might any of the tests be experienced as invasive or culturally insensitive?*

To our knowledge, none of the tests was deemed as invasive or culturally insensitive.

c) *How did you determine that work was sensitive to traditions, restrictions, and considerations of all cultural and religious groups in the study population?*

Ahead of the study, the proposed activities were discussed with colleagues at Jinja District Council and with community leaders and Village Health Teams from Buwenge subcounty. In addition, we engaged teachers and parents in planning the detailed standard operating procedures for the study. Prior to recruitment we held meetings to discuss the proposed work with teachers, parents and students and to address their questions. As well, meetings with village leaders and village members were held to discuss the study. Through these approaches, we ensured that the work was sensitive and considerate to the different groups among the study population.

d) *Were biowaste and radioactive waste disposed of in accordance with local laws?*

Waste from laboratory and clinical procedures was disposed of according to approved safety guidelines provided by the MRC/UVRI and LSHTM Uganda Research Unit, and by the Uganda Virus Research Institute.

e) *Were any structures built that would have impacted members of the community or the environment (such as handwashing facilities in a public space)? If so, how did you ensure that you had appropriate community buy-in?*

We modified an already existing laboratory space at Buwenge District Hospital with solar system and project specific laboratory equipment. We held consultations with the hospital administration during this process to address any concerns and seek their input. Following the end of the study, we handed over the solar system to the hospital administration.

f) *How might the study have impacted existing health-care resources (such as staff workloads, use of equipment that is typically employed elsewhere, or reallocation of public funds)?*

Our study had no or minimal impact on existing health-care resources, as all necessary resources were provided through the study funding.

8. Finally, please provide the title (eg, Dr/Prof, Mr/Mrs/Ms/Mx), name, and email address of an author who can be contacted about this statement. This can be the corresponding author.

Name: Dr Gyaviira Nkurunungi
Email: Gyaviira.Nkurunungi@mrcuganda.org
